# Pushing the analytical limits: new insights into complex mixtures using mass spectra segments of constant ultrahigh resolving power†
†Electronic supplementary information (ESI) available. See DOI: 10.1039/c9sc02903f


**DOI:** 10.1039/c9sc02903f

**Published:** 2019-07-05

**Authors:** Diana Catalina Palacio Lozano, Remy Gavard, Juan P. Arenas-Diaz, Mary J. Thomas, David D. Stranz, Enrique Mejía-Ospino, Alexander Guzman, Simon E. F. Spencer, David Rossell, Mark P. Barrow

**Affiliations:** a Department of Chemistry , University of Warwick , Coventry , CV4 7AL , UK . Email: M.P.Barrow@warwick.ac.uk; b Department of Chemistry , Universidad Industrial de Santander , Bucaramanga , Colombia; c Molecular Analytical Science Centre of Doctoral Training , University of Warwick , Coventry , CV4 7AL , UK; d Sierra Analytics Inc. , Modesto , California , USA; e Instituto Colombiano del Petróleo , Ecopetrol , Piedecuesta , Colombia; f Department of Statistics , University of Warwick , Coventry , CV4 7AL , UK; g Department of Economics & Business , Universitat Pompeu Fabra , Barcelona 08005 , Spain

## Abstract

A new strategy has been developed for characterization of the most challenging complex mixtures to date, using a combination of custom-designed experiments and a new data pre-processing algorithm. In contrast to traditional methods, the approach enables operation of Fourier transform ion cyclotron resonance mass spectrometry (FT-ICR MS) with constant ultrahigh resolution at hitherto inaccessible levels (approximately 3 million FWHM, independent of *m*/*z*). The approach, referred to as OCULAR, makes it possible to analyze samples that were previously too complex, even for high field FT-ICR MS instrumentation. Previous FT-ICR MS studies have typically spanned a broad mass range with decreasing resolving power (inversely proportional to *m*/*z*) or have used a single, very narrow *m*/*z* range to produce data of enhanced resolving power; both methods are of limited effectiveness for complex mixtures spanning a broad mass range, however. To illustrate the enhanced performance due to OCULAR, we show how a record number of unique molecular formulae (244 779 elemental compositions) can be assigned in a single, non-distillable petroleum fraction without the aid of chromatography or dissociation (MS/MS) experiments. The method is equally applicable to other areas of research, can be used with both high field and low field FT-ICR MS instruments to enhance their performance, and represents a step-change in the ability to analyze highly complex samples.

## Abstract 2

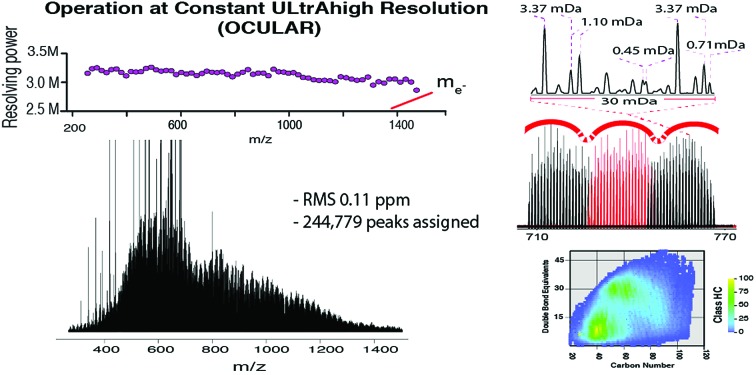
A new strategy has been developed for characterization of the most challenging complex mixtures to date, using a combination of custom-designed experiments and a new data pre-processing algorithm.

## Introduction

### Ultrahigh resolution mass spectrometry

Mass spectrometry has been increasingly used to make significant strides in the characterization of highly complex samples, affording the ability to establish detailed molecular compositions of samples previously considered too challenging. Such sample characterization requires measurements of ultrahigh resolving power and high mass accuracy in order to minimize the possibilities of unresolved peaks and misassignments.^1^–^3^ To date, Fourier transform ion cyclotron resonance mass spectrometry (FT-ICR MS) has represented the state-of-the-art, offering the highest performance in terms of both resolving power and mass accuracy^4^,^5^ and providing abilities to resolve components of complex mixtures without any prior fractionation and to assign molecular formulae with sub-ppm mass accuracy. This has been demonstrated with respect to applications such as natural organic matter,^6^–^11^ metabolomics,^12^–^14^ proteomics,^15^,^16^ and petroleum-related samples, now known as petroleomics,^17^–^27^ amongst others. Whilst significant progress has been made in these fields, challenges remain.

Resolving power at full width half maximum (FWHM) is defined as *m*/Δ*m*_50%_, where *m* is the mass of the ion being measured and Δ*m*_50%_ is the full width of a spectral peak at half-maximum peak height; higher resolving power affords the ability of the mass spectrometer to separate compositions with small mass differences. For high field FT-ICR instruments, resolution over 400 000 FWHM at *m*/*z* 400 can be routinely achieved when magnitude mode broadband mass spectra of petroleum samples are acquired; Cho *et al.* have recently compiled a list of the current literature, citing resolving powers achieved using FT-ICR MS.^16^ For analysis of petroleum samples, ultrahigh resolution affords the ability to resolve species differing in mass by 3.37 mDa, for example, which corresponds to a common compositional difference between ^12^C_3_ and ^32^S_1_H_4_. Mass error is typically measured in parts per million^28^ (ppm), where smaller values indicate a higher confidence in the assignment of a given molecular formula.^29^,^30^ Whilst FT-ICR MS is uniquely well-suited for the molecular characterization of highly complex mixtures,^31^ there are many experimental challenges which influence resolving power, mass accuracy, dynamic range, and maximum number of peaks that can be detected. As an example, for a given FT-ICR mass spectrometer (constant magnetic field), it is known that the mass resolving power is inversely proportional to *m*/*z*.^29^,^30^ That is, for a broadband mass spectrum, spanning a broad *m*/*z* range, the resolving power decreases significantly with increasing *m*/*z*. For complex mixture analysis, resolving power must be maximized to avoid loss of information and mass errors must be minimized to have confidence in the molecular formula assignments. As a result, the most challenging samples require pushing the analytical limits of current methods.

### Complex mixtures

One of the most direct ways to increase the resolving power is to use higher magnetic fields, but larger magnets also increase instrument cost significantly.^30^ Resolution increases proportionally with the signal acquisition time, which is determined by the data set size divided by the sampling frequency.^29^,^30^ Thus, another means for increasing the resolving power is to acquire longer time domain data, if the data acquisition system and space-charge effects permit. Similarly, it has been demonstrated that resolving power can be enhanced by up to a factor of 2 when the raw data is “phased” to produce absorption-mode data, instead of using the more traditional magnitude mode.^32^–^34^ For example, in 2014,^35^ absorption mode data was used to establish world records using an underwater asphalt volcano sample: a resolving power of 1 400 000 FWHM was demonstrated at *m*/*z* 515, with 85 920 molecular compositions assigned. In early 2018, two 21 T FT-ICR mass spectrometers in different laboratories were applied to the analysis of complex mixtures; one achieved a resolution of approximately 1 000 000 FWHM at *m*/*z* 2700 using absorption mode (6 s time-domain transient)^36^ and the other a resolving power of 2 700 000 FWHM at *m*/*z* 400 using absorption mode (transient length of 6.3 s), with an accompanying assignment of 49 000 molecular compositions.^37^

Accurate mass measurements require sufficiently high resolving power to ensure peaks are well resolved. Furthermore, it is beneficial to trap low ion populations during detection in order to minimize coulombic repulsion, which gives rise to so-called “space-charge effects,” which can affect both FT-ICR and Orbitrap mass spectrometers. Space-charge effects can include peak coalescence, decreased resolution, distorted peak shapes, frequency shifts, and the “spontaneous loss of coherence catastrophe,” amongst others.^38^–^42^ Whilst lowering ion populations may reduce space-charge effects,^43^ a minimum number of ions in a cloud is needed for ion cloud stabilization and an order of 10^6^ singly-charged ions is the upper capacity limit of a modern ICR cell.^40^ As a result, the balance between the minimum number of ions required per peak and the maximum number of trapped ions means that there are consequences for dynamic range and therefore for the maximum number of peaks detected within a single FT-ICR mass spectrum. Additionally, where FT-ICR instruments may employ an ion trap or collision cell for ion accumulation prior to transfer to the ICR cell, coulombic effects can result in discrimination effects^44^–^47^ (such as loss of low mass ions, for example), leading to a shift of the mass distribution once the ions have been transferred to the ICR cell. The number of ions involved in a single experiment therefore has consequences both for the use of ion traps or collision cells, as well as for the excitation and detection within the ICR cell. These effects are of greatest relevance when studying highly complex samples and can be addressed in part by reducing the concentration of sample solutions, reducing the syringe pump flow rate when directly infusing the sample into the mass spectrometer, or reducing the number of ions accumulated at the front end of the instrument (typically by reducing the ion accumulation time), prior to transmission to the ICR cell. More accurate mass measurements can also be attained if a separate calibration equation is applied to small segments of the broadband spectra,^48^ known as a “walking calibration.”

Further experimental methods can be employed to attempt to overcome these space-charge limitations. Reduction of the number of ions accumulated will reduce space-charge effects, but such a method can adversely affect dynamic range, so that many species of lower abundance cannot be detected. As a consequence of the optimization of the number of ions transferred to the ICR cell, however, there are limitations in relative-quantitative analysis of the measurements. To overcome this, a “spectral stitching” method was carried out by Southam *et al.* in 2007 to improve metabolome coverage and mass accuracy (mass error of 0.18 ppm), and the method was later used for further work in metabolomics and lipidomics.^13^,^49^ In a stitched mass spectrum, a mass filter such as a quadrupole is used to selectively accumulate ions for a succession of narrow *m*/*z* windows, producing individual mass spectra for each *m*/*z* range. These segments are then combined to generate a mass spectrum spanning the full *m*/*z* range of interest. Gaspar *et al.*^50^ applied spectral stitching to the analysis of crude oil, which was also adapted by Al-Jawad *et al.*^51^ In his work, Gaspar demonstrated that it was possible to detect more peaks using this method, compared with a traditional broadband mass spectrum, where an entire mass range is detected in a single experiment. A negative effect, though, was a significant change in the mass envelope of the crude oil in the stitched spectrum, compared with the broadband data. Similarly to FT-ICR MS experiments, Vetere *et al.*^52^ have shown that the spectral stitching method using an Orbitrap mass spectrometer increases the coverage of crude oil composition, but the resolution of the instrument was not enough to baseline resolve peaks with a mass difference of 3.37 mDa beyond *m*/*z* 450 with a transient length of 1.53 s.

Similar improvements in performance were observed in late 2017 by Krajewski *et al.*, where a record number of 126 264 unique elemental compositions including isotopologues (or 67 237 monoisotopic assignments) were assigned when applying a spectral stitching method to the characterization of an asphalt volcano sample using FT-ICR mass spectrometry.^53^ The instrument parameters were tuned for individual mass segments and this influenced the overall mass envelope of the stitched spectrum, compared to a broadband acquisition. Using the spectral stitching method, a resolution of approximately 1 800 000 FWHM was achieved at *m*/*z* 300; within the same data, a resolving power of approximately 200 000 FWHM was measured at *m*/*z* 1100, due to the decrease in resolving power with increasing *m*/*z*. It was no longer viable to resolve and assign the compositions at this mass and above due to the relationship between resolving power and *m*/*z*, which is a well-known and recurring performance limitation. Mass measurement accuracy was improved only at lower masses, however, potentially due to the decreasing ability to sufficiently resolve peaks at higher mass.

### OCULAR

In this work, we demonstrate a new strategy for complex mixture analysis where segments of near constant ultrahigh resolving power of an order of millions (typically >3 000 000 FWHM) across a broad *m*/*z* range (*m*/*z* 260–1500) were acquired, processed, and stitched. The method consists of a modified stitching method which: uses increasing time domain data length with increasing *m*/*z*, uses phasing to produce absorption mode data segments, and incorporates the development of software that determines the best position for overlapping the many segments, corrects the relative abundances of the ions in the segments (attenuated during the mass filtering process), and then automatically stitches the segments together. For brevity, this method will be referred to as the operation at constant ultrahigh resolution (OCULAR) method. The method was applied to a distillable fraction of a petroleum sample as a proof of concept and then to a *truly non-distillable* fraction of a vacuum residue of a petroleum sample, which had never been successfully characterized previously, due to its complexity. A new record of 244 779 assignments has been achieved, while using a 12 T FT-ICR MS instead of the very highest field currently available (15 T or 21 T), illustrating the performance enhancements afforded by OCULAR; use of the approach with even higher magnetic fields would be expected to offer even further improvements in performance. OCULAR affords the ability to operate at approximately constant resolving powers across broad *m*/*z* ranges in contrast to other methods, reduces distortion of mass envelopes, improves dynamic range, increases the number of peaks detected, and effectively overcomes the well-known decrease in ability to resolve peaks and assign molecular formulae at higher end of a given *m*/*z* range. While petroleum samples have been used here due to their complexity, the OCULAR method is of course applicable to other research areas, affording the ability to characterize complex samples where traditional broadband mass spectra will not suffice. The OCULAR method pushes the current analytical limits of the highest performance mass spectrometers and can extend the performance of lower resolution instruments, enhancing their capabilities for application to many fields.

## Experimental methods

### Sample preparation

Two fractions obtained from a South American vacuum residue were selected for characterization. The vacuum residue has a density of 1.0251 g cm^–3^ and 4.9 wt% of asphaltenes. The lightest fraction was obtained by supercritical fluid extraction and was chosen as the test bed for the new method. Approximately 90% of the sample constituents have boiling temperatures lower than 720 °C, atmospheric equivalent temperature (AET), and thus, it is identified as a distillable fraction (D-F). The second fraction analyzed in this work is the maltenes of a truly non-distillable fraction (ND-F) and corresponds to the residue obtained by molecular distillation. Thus far, the ND-F sample had not been characterized successfully due to its complexity, despite a wide range of sample preparation methods and mass spectrometric experiment types having been conducted. The D-F sample was used for method development, prior to application to the ND-F sample for its first successful ultrahigh resolution characterization. The true boiling point curve and more detailed information of these samples can be found in the ESI (see Fig. S1 and Table S1†). The samples D-F and ND-F were diluted in high performance liquid chromatography (HPLC) grade toluene (Fisher Scientific, Loughborough, U.K.) at concentrations of 0.05 and 0.04 mg mL^–1^, respectively. It has been recommended previously that concentrations of 0.05 mg mL^–1^ or lower are used because aggregation of many heavy petroleum components, such as through pi–pi stacking of aromatic rings, occurs at higher concentrations.^54^–^56^

### OCULAR workflow

It is well-known that the resolving power for an FT-ICR mass spectrometer is proportional to the magnetic field and the acquisition time, and is inversely proportional to the *m*/*z* (see more details in ESI†). Additionally, there is a finite number of ions that can be stored in an ICR cell and large numbers of ions lead to space-charge effects, such as peak coalescence and degradation of resolving power and mass accuracy.^40^ Higher resolving power and reduction of space-charge effects become more important as the sample complexity increases (*e.g.* multiple, small differences between *m*/*z* values within a broad mass range). The resolving power increases linearly with the acquisition time. The acquisition time, in turn, is proportional to data set size and inversely proportional to the sampling frequency (defined as twice the frequency of the lowest *m*/*z* being detected, in accordance with the Nyquist theorem). As a result, the resolving power increases with the low *m*/*z* limit of detection (higher *m*/*z* cut-off). Space-charge effects can be reduced by acquiring mass spectra of narrower *m*/*z* segments, therefore reducing the total number of ions per experiment, and then combining the segments to produce the complete data set, spanning a full *m*/*z* range. The OCULAR method additionally uses segments with increasing low *m*/*z* cut-offs (longer acquisition times) to maintain near constant resolving power and to reduce space-charge effects (see details in Fig. S2 in ESI†).

**Fig. 1:**
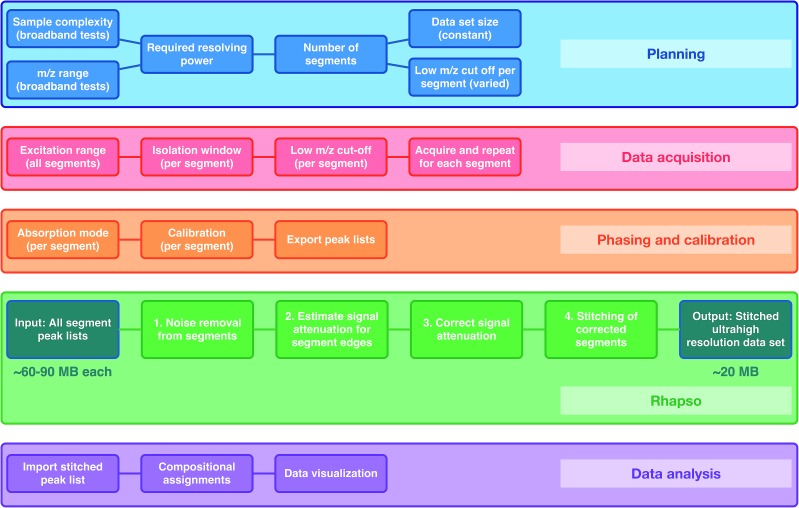
Flowchart describing the OCULAR method. During the planning stage, the sample complexity, *m*/*z* range, and, therefore, required number of segments are determined. The desired resolution then determines the number of segments, data set size, and the low *m*/*z* cut-off per segment. During the acquisitions of data, a quadrupole is used for isolation windows, the low *m*/*z* cut-off for detection is increased for sequential segments. During the third stage, the mass spectra are phased and calibrated, and peak lists are exported using a low signal-to-noise threshold. The peak lists are processed using Rhapso and the resulting, complete data set has a smaller file size than the individual segments, due to automated noise removal. The final data set is exported for compositional assignments and data visualization.

The workflow can be considered in five stages: *Planning*; where the required resolving power, data set size, and number of segments are defined; *Data acquisition; Phasing and calibration*; *Rhapso* (in-house software) for automated stitching of the data segments; and *Data analysis* of the stitched mass spectrum. A flowchart of the method described below can be found in Fig. 1.

#### Planning

The number of segments is defined by the sample complexity (dynamic range and resolving power), the total *m*/*z* range, and the required resolving power. Multiple, closely-spaced mass splits are often characteristic of complex samples and can act as a guide for the resolving power that is required. In addition, the desired resolving power determines the data set size and the low *m*/*z* cut offs of the segments. For the following experiments, a target resolution was chosen for the lightest sample (D-F) and the heaviest sample (ND-F), using 4 M and 8 M data set sizes, respectively. The low *m*/*z* cut-offs were re-calculated‡ for each segment in order to maintain constant resolving power across the entire *m*/*z* range for the stitched mass spectrum (see details in Table S3 in the ESI† and see the Notes and references section).

#### Data acquisition

The samples were ionized with an APPI II (Bruker Daltonik GmbH, Bremen, Germany) atmospheric pressure photoionization (APPI) source. Diluted samples were directly injected at a flow rate of 500 μL h^–1^, using a drying gas temperature of 250 °C, vaporizer temperature of 350 °C, and capillary potential of 1200 V. The ion source was coupled with a 12 T solariX (Bruker Daltonik GmbH, Bremen, Germany) FT-ICR mass spectrometer, equipped with an Infinity Cell. The instrument was operated using solariXcontrol (Bruker Daltonics, Billerica, MA, USA). The ions were accumulated in a collision cell prior to being transferred to the ICR cell, where potentials of 0.4 V were applied to the front and back trap plates in order to trap the ions for detection. A broadband mass spectrum of the D-F sample was obtained using data size of 4 M with a detection range of *m*/*z* 250–3000 (acquisition time of 2.31 s), and a total of 100 time-domain transients were co-added. For the stitched mass spectra, a quadrupole was used to transmit narrow *m*/*z* ranges (*e.g.* specifying *m*/*z* widths of 24) to the collision cell for accumulation, prior to transfer to the ICR cell. The detection ranges for each segment were changed, while, in contrast, the ion accumulation time and the excitation range and magnitude were kept constant for each segment in order to avoid influencing the peak abundances and therefore the overall mass envelope. To improve the detection of low abundance components, a higher ion accumulation time was used for all segments, compared to broadband mass spectra (where space-charge effects would be deleterious). The stitched mass spectrum of the D-F sample was acquired by segmenting the mass range using windows each with an *m*/*z* width of 24, where each segment was a 4 M data set and resulted from the co-addition of 50 scans. A final mass spectrum was obtained by the stitching of 41 segments which spanned the *m*/*z* range of interest. By contrast, for the significantly more complex ND-F sample, the stitched mass spectrum was produced using 65 windows, each using an *m*/*z* width of 24, but where the segments were acquired as 8 M data sets and 100 scans were co-added. A summary of the acquisition parameters can be found in Table S4 (see ESI†).

#### Phasing and calibration

The acquisition method was externally calibrated in the mass range of *m*/*z* 300–800 using a petroleum sample. The offline phasing of data to produce absorption-mode mass spectra was performed using FTMS Processing 2.1.0 with an asymmetric apodization (“Kilgour”) function.^57^ The mass spectra were internally recalibrated after phasing. The broadband spectrum was recalibrated using an abundant homologous series (S_1_ class, 9 double bond equivalents), with peaks spaced by 14.01565 Da (*i.e.* CH_2_ units). For the OCULAR experiment, the segmented windows were recalibrated using the HC class with a mass difference of 2.01565 Da (*i.e.* H_2_ unit), due to the narrower *m*/*z* range available per segment. This calibration used a peak per two daltons, with up to twelve peaks within each 24 Da window.

#### Rhapso

Using the OCULAR method, multiple, overlapping, ultrahigh resolution windows, each of a narrow *m*/*z* range, are combined to produce the mass spectrum spanning the full *m*/*z* range of interest. The isolation of ions of a specific *m*/*z* range to produce a segment typically leads to an attenuation in signal at the low *m*/*z* and high *m*/*z* edges of the given window. Also, it is known that isolation of ions using a quadrupole can be less efficient at higher *m*/*z*, causing low-abundance signals to occasionally be observed outside the upper *m*/*z* limit of the isolation window.^58^ To address these effects, the calibrated peak lists of each absorption mode segment were exported, stitched, and corrected using a novel algorithm called “Rhapso,” named after a character from Greek mythology, known for stitching or sewing.

Rhapso consists of four steps, as represented in the flowchart shown in Fig. 1 and demonstrated in Fig. S4 and S5.† The algorithm trims each segment at the high and low *m*/*z* ends of the observed signal to prevent the inclusion of peaks outside of the isolated window, producing reduced-width segments. The signal attenuation is then measured across the segments and compensation is applied. The multiple, corrected, reduced-width segments, which span the *m*/*z* range of interest in total, can then be combined appropriately after determining the suitable regions for overlap, resulting in the stitched mass spectrum. The combination of the experimental methodology (producing segments of constant ultrahigh resolving power, amongst other parameters) and data processing (trimming, correcting, and appropriately stitching the many segments) form the basis of the OCULAR method.

#### Data analysis

For the assignment of molecular compositions, the software Composer version 1.5.7 (Sierra Analytics Inc., Modesto, CA, USA) was used. Each elemental composition was assigned with a maximum of C_200_, H_1000_, N_4_, S_3_, O_5_, V_1_, and the maximum number of double bond equivalents (DBE) was selected as 60. The maximum mass errors for the broadband mass spectrum was set to 1 ppm, while values of 0.45–0.50 ppm were used for the stitched data, due to the reduced mass errors when using the OCULAR method.

## Results and discussion

Petroleum has been described as “nature's most complex mixture,” and therefore petroleum samples present some of the greatest analytical challenges. As a result, we have used such samples to develop and assess the OCULAR method, although the method is equally suitable for other applications. Typically, 40–60% of components in heavy crude oils evaporate above 540 °C (540+ °C).^59^ This fraction is known as vacuum residue (VR), and is characterised by its high complexity and heteroatomic content (N, S, O and traces of metals). While FT-ICR MS is well-known to be an extremely versatile technique, uniquely suited to the detailed molecular characterization of complex samples,^26^,^27^,^60^ it has been demonstrated that a more complete characterization of the composition of heavy ends of petroleum can be afforded by the prior separation or fractionation of the sample.^61^ Due to the low volatility of the components of vacuum residues, however, they cannot be chromatographically separated: they are not amenable to gas chromatography (GC)^62^,^63^ or liquid chromatography (LC).^64^ In addition, heavy or polar compounds can be irreversibly adsorbed on LC or GC columns, leading to deterioration of the chromatographic performance and to incomplete elution of the analytes.^65^,^66^ For such samples, separation by supercritical fluid extraction (SFE) (by solubility) or by molecular distillation (by boiling point) is viable.^67^,^68^ The analysis of fractions of vacuum residues by SFE FT-ICR MS has been attempted previously,^68^,^69^ but compositional analysis was not achieved due to the low abundance of the mass spectra obtained for the end-cut.^69^ Fractions obtained by molecular distillation have been analyzed by high resolution mass spectrometry (resolving power approximately 14 000 FWHM),^70^ but a detailed compositional analysis by FT-ICR MS has thus far remained elusive. The sample complexity presents analytical challenges due to the limited number of ions that can be trapped without overfilling the collision cell or the ICR cell. The number of trapped ions that limits peak coalescence effects depends linearly with the magnetic field and the mass difference of the ions, and is inversely proportional to the square of the mass.^71^ Thus, species with small mass differences and at higher masses are more strongly affected by this phenomenon. Spectral stitching can address these limitations and, in contrast to previous examples of data stitching, the OCULAR method reduces distortion of the *m*/*z* envelope by not re-tuning for maximum signal abundance per segment, reduces signal attenuation and determines optimum regions for stitching through use of an algorithm, and, through the use of carefully controlled parameters, produces data of constant resolving power of greater than 10^6^ (*e.g.* approximately 3 000 000 FWHM) across a broad *m*/*z* range.

### Proof of concept

**Fig. 2:**
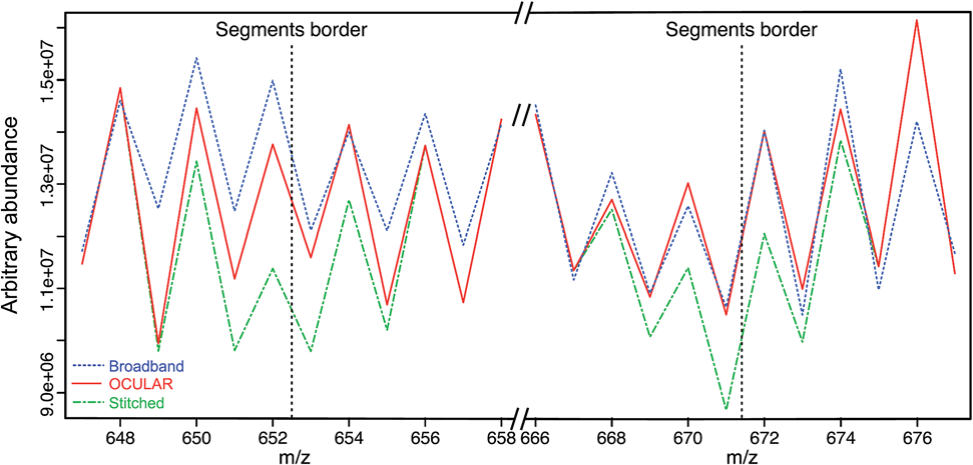
Mean abundance of clusters of peaks at each nominal *m*/*z*, using an example of a segment centred at *m*/*z* 662 and the edges of two, adjacent segments. The broadband spectra (blue), stitched spectrum without abundance correction (green), and stitched spectrum with abundance correction (red) are compared. It can be seen that using the OCULAR method ensures the abundances are closer to those observed under a typical broadband experiment, as signal attenuation during the segmentation process is addressed.

To examine the suitability of the new method, a light fraction of a vacuum residue (the distillable fraction or “D-F” sample) was analyzed using the OCULAR method and the results compared with those obtained *via* a traditional, broadband mass spectrum. Fig. 2 shows the average abundance of the clusters of peaks at each nominal *m*/*z* for three segments: a segment centred at *m*/*z* 662 and the edges of two, adjacent segments. Due to the need to control the total ion population, the signal abundance was lower during broadband acquisition. Higher ion accumulation times can be used when using a segmented approach, increasing dynamic range. For the comparison in Fig. 2, the abundances of the peaks within broadband data were rescaled using the mean difference for the stitched spectra over two peak clusters where no abundance correction was applied. It can be seen that the abundance correction reduces the signal attenuation at the lower and higher edges of each segment; other undulations throughout the mass range are primarily due to the natural patterns within petroleum samples (see Fig. S5†). The lower abundance regions consistently showed the greatest discrepancy when compared to the broadband data. This is another effect of using isolation windows, as the observation of more peaks and of lower abundance peaks, sometimes previously below detection thresholds, can cause a lower average abundance.

**Fig. 3:**
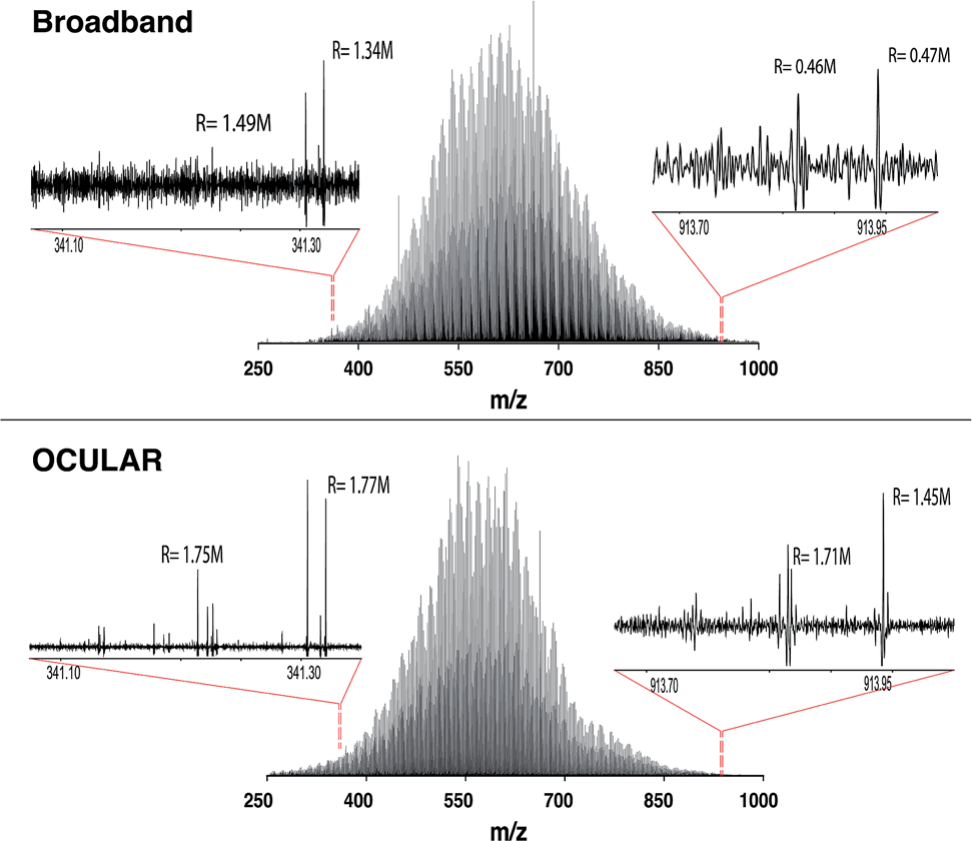
Mass spectra of the distillate fraction of crude oil (D-F). The top shows the broadband mass spectrum, while the bottom mass spectrum represents use of the OCULAR method by the stitching of 42 windows, each with an *m*/*z* width of 20. The approximately constant resolving power (labelled R) of the OCULAR data can be observed in the enlarged regions and contrasted with the resolving powers shown for the broadband data.

As shown in Fig. 3, a similar overall mass distribution between the broadband and the stitched mass spectrum was attained. This demonstrates that keeping the experimental parameters constant when using the OCULAR method, rather than tuning to maximize signal abundance per segment, results in a smooth mass envelope which is more reflective of the composition of the sample. The mean molecular weight of the broadband spectra was shifted towards higher *m*/*z*, however, as a consequence of space-charge effects experienced within the collision cell prior to transfer to the ICR cell,^44^,^47^,^72^ even with the low sample concentration and low ion accumulation time used for the acquisition (see Table S4†). The overall *m*/*z* range was similar for both methods, but a significant number of peaks with greater signal-to-noise ratio (S/N) were detected at lower and higher *m*/*z* regions of the stitched spectrum (see Fig. 3 and S8†).

It can also be seen that peaks at the high *m*/*z* end of the mass distribution were observed to have higher resolving power and higher S/N in the data produced using the OCULAR method. The mean resolving power per segment was almost constant (average of 1 792 000 FWHM) over the full mass range, while the measured resolving power of the broadband mass spectrum, in both magnitude and absorption-mode, decreased with *m*/*z* in the expected manner. The decrease in resolution with *m*/*z* normally associated with FT-ICR mass spectra was avoided by a steady increase of the transient length with each segment: 2.3 s for the window isolated at *m*/*z* 260 and up to 9.6 s at *m*/*z* 1002, for example. As a result, the resolving power at *m*/*z* 945, for example, was increased by a factor of 3.2 and 5.6 when comparing the OCULAR data with broadband data in absorption mode and magnitude mode, respectively.

**Fig. 4:**
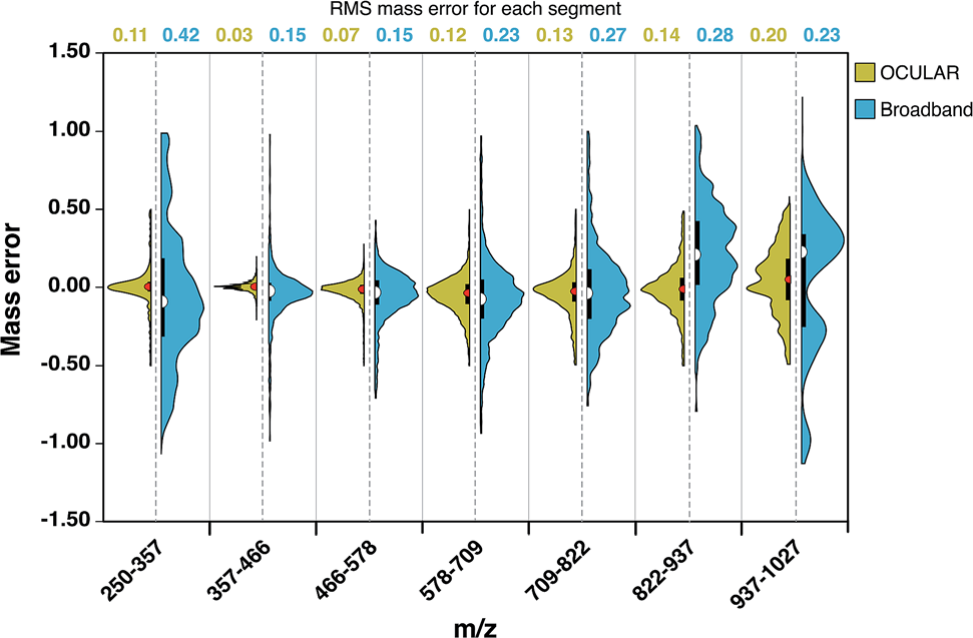
Half violin plots for stitched and broadband mass spectra, comparing the range of mass errors (in ppm) associated with molecular formulae assignments *versus m*/*z*. The left-hand sides, in green, indicate the data obtained for the OCULAR data, while the right-hand sides, in blue, display the data from the broadband spectrum. The values at the top indicates the RMS mass error, in ppm, for each *m*/*z* range.

As a result of the ultrahigh resolution of the stitched spectra and the improved dynamic range, 97.8% of the peaks were assigned within 0.5 ppm in the OCULAR mass spectrum, compared with 91.2% of peaks being assigned within 1 ppm for the broadband mass spectrum. In Fig. 4, the distribution and probability densities of the mass errors are shown for different regions of the mass spectra. The blue violin diagrams (right-side) correspond to the broadband mass spectrum and the green violin diagrams correspond to the OCULAR method. The red and white dots within the plot represent the medians of the data, while the black bars in the centres indicate the interquartile range of the mass errors. The spread of the mass errors remained relatively consistent with *m*/*z* when using the OCULAR data, while the broadband data displayed a trend of increasing mass error with *m*/*z*.

For the broadband data, the mass error is dispersed ±1 ppm above *m*/*z* 822 and below *m*/*z* 357. The median value and the dispersion of the data at high mass is shifted toward positive values, which indicates a shift of the detected frequencies towards lower values as a consequence of space-charge effects. In contrast, the error distribution for the OCULAR data indicates that a high percentage of the mass errors is concentrated around the median, which is closer to 0.11 ppm. This figure demonstrates that the mass accuracy was noticeably improved in the full-mass range in the combined spectra. On average, the RMS mass error was improved by approximately a factor of 2, decreasing from 0.23 ppm for the broadband mass spectrum to 0.11 ppm for the OCULAR mass spectrum. Thus, the new method has afforded the ability to detect a greater number of peaks and assign molecular formulae with increased confidence, based on improved resolving power and dynamic range, aiding accurate mass measurements and observation of fine isotopic distributions. A greater detail of the results obtained for the D-F sample can be found in the ESI† (Section 3). At higher masses and for very complex samples, exhibiting large numbers of peaks of very small mass differences, ultrahigh resolving power is essential for unambiguous assignments of molecular formulae. Here, it becomes necessary to maximize the performance of the instrumentation and experimental methodologies.

### Non-distillable fraction

According to Boduszynski, due to the broad molecular weight, high heteroatomic content, and low volatility, “the truly nondistillable residues are by far the most difficult to analyze.”^73^ When the maltenes of the ND-F of the South American vacuum residue were analyzed using broadband experiments, a mass spectrum could not be obtained (see Fig. S14†), despite a variety of sample preparation and instrument tuning methods. A simple experiment was performed using electrospray ionization (ESI), which selectively ionizes polar components and so produces less complex data,^74^ in order to demonstrate that the challenge of characterizing this sample is not due to low ionization efficiencies of its components, but due to a space-charge effects resulting from overfilling the collision cell or ICR cell. Mass spectra could be obtained when using an isolation window *m*/*z* width lower than 300, but no signal was detected when the isolation window *m*/*z* width was 600 or higher, however. It is therefore clear that no signal would be observed when attempting traditional broadband experiments, regardless of sample preparation methods or instrument tuning. As the window size was increased, peaks with small mass differences began to coalesce and, thus, the number of peaks detected decreased. The ESI experiment demonstrated that the sample components could indeed be ionized and that the sample complexity would lead to a number of space-charge effects. Ion loss and mass discrimination can occur in the collision cell, while the minimum number of required ions per peak and the density of peaks per nominal *m*/*z* meant that the ICR cell would be rapidly overfilled when attempting to have sufficient signal. As a comparison, the same experiment was performed using the D-F sample (Fig. S15†), which would be expected to be significantly less complex. In contrast to the ND-F sample, signal was not lost when expanding the isolation window size, but space-charge effects such as peak coalescence and frequency shifts were observed when broad ranges were used. Although the isolated mass was centred at *m*/*z* 500, the mass distribution was increasingly shifted toward higher mass, as a function of window width, due to displacement of ions when overfilling the collision cell. This observation is consistent with the proposed explanation for the shift of the molecular weight distribution observed in the broadband spectra of the DF sample (Fig. 3) and indicates that the mass distribution obtained in the stitched mass spectrum more accurately represents the molecular distribution of the sample.

**Fig. 5:**
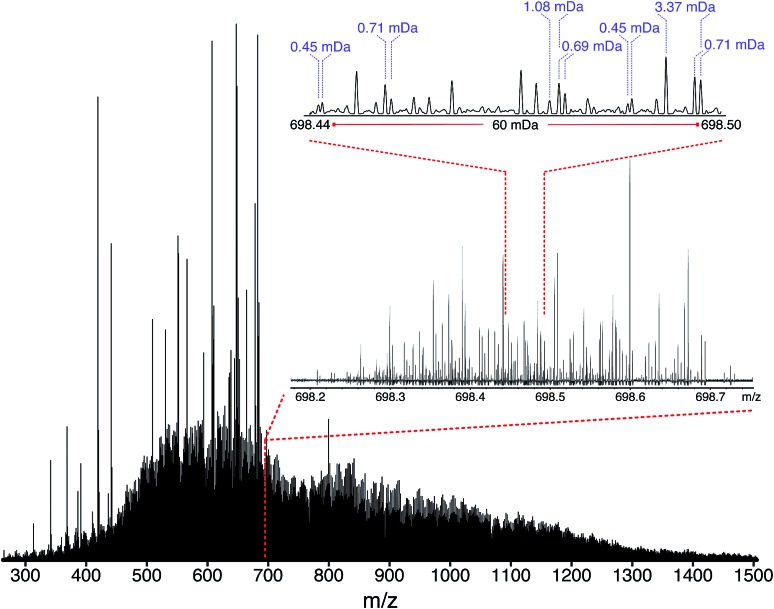
Stitched mass spectrum of the ND-F sample, with peaks spanning *m*/*z* 260–1505. The spectrum was obtained by stitching 65 windows, each with an *m*/*z* width of 24, with steadily increasing acquisition time per segment to result in near constant resolving power across the *m*/*z* range. An enlarged region, showing assignments for *m*/*z* 698, is shown.

The mass spectrum of the maltenes of ND-F was obtained for the first time using the OCULAR method, following refinement using the D-F sample. In order to reduce the probability of aggregation of components, such as *via* pi–pi stacking of rings, the ND-F sample was dissolved at a concentration of 0.04 mg mL^–1^, lower than typically cited for asphaltene analysis.^75^–^77^ Due to the concentration being so low, however, background peaks from solvent contaminants can be found in proportionally high abundance, such as shown in the isolation window centred at *m*/*z* 605 (Fig. S16†). Despite this, the mass distribution, mass accuracy, and resolving power were not significantly affected. The extraordinary complexity of the sample is illustrated in Fig. 5 (further examples of enlarged segments can be seen in Fig. S17–S19†). Peaks separated by mass differences less than the mass of an electron (0.00054858 Da), were regularly found throughout the mass spectrum. The complexity of the composition at each nominal *m*/*z* increases with *m*/*z*, although there is a trade-off with signal abundance (see Fig. S19†). In Fig. 5, the mass distribution spans *m*/*z* 260–1505, with a mean molecular weight of 890 Da. Due to the low mass cut-off that this range requires, in turn determining the acquisition time, a traditional broadband mass spectrum would not be sufficient to resolve the well-known 1.10 mDa separation for approximately the half of the mass range; the observed separations of less than the mass of an electron would clearly be impossible to resolve and information would be lost. Even when using the OCULAR method, the higher mass range proved challenging: peaks could be observed between *m*/*z* 1500–1800 but sufficient signal abundance could not be acquired to produce viable segments for inclusion. The mass range maltene fraction of the ND-F of the VR, was consistent with the broad mass distribution predicted by Boduszynski for the non-distillable fractions of crude oils, where compounds were anticipated up to *m*/*z* 2000.^73^,^78^

**Fig. 6:**
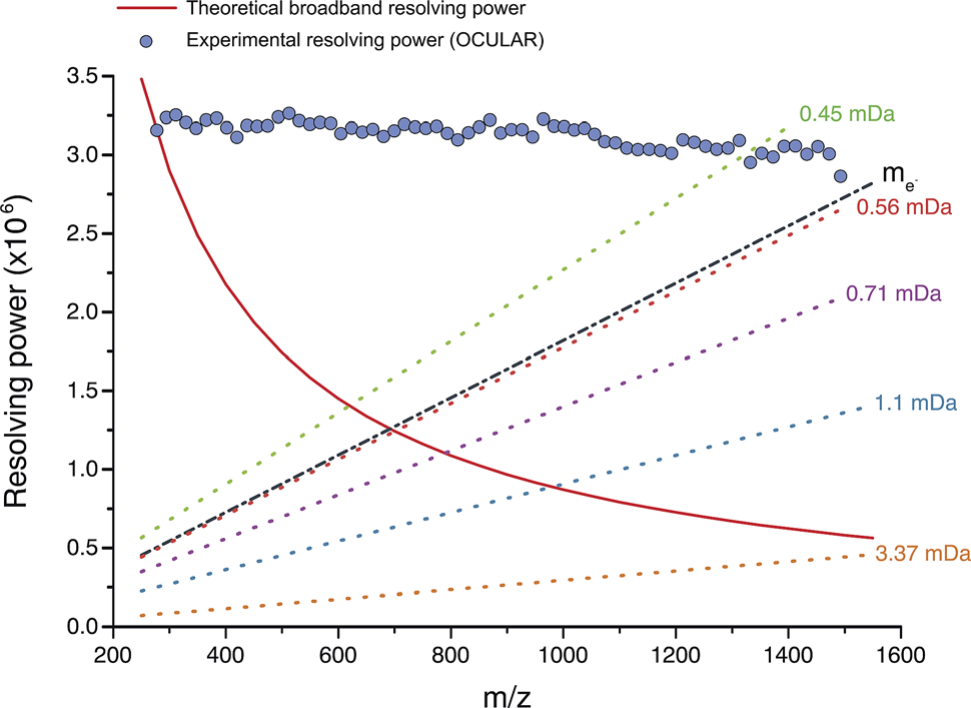
Red line: mass resolving power calculated for a mass spectrum acquired in broadband mode at 8 M using a 12 T FT-ICR MS (in similarity to the OCULAR data) with a low mass cut-off of *m*/*z* 239.5, without apodization, and obtained under ideal conditions, such as a perfect vacuum and no space-charge effects.^29^ The dotted lines represent the calculated minimum mass resolving powers required to resolve two peaks of comparable abundance, separated by the mass difference listed. The data points mark the mean experimental resolving power per 20 Da window. The resolving power afforded by the OCULAR method is sufficient to resolve peaks separated by a difference equivalent to only the mass of one electron (*m*_e_, continuous black line; 0.0005485 Da) across the full mass range.

The mean resolving power per adjusted segment (*m*/*z* width of 20) is shown in Fig. 6. Due to determination in advance of the required acquisition times for each of the 65 segments, the mass resolving power was essentially constant at approximately 3 million FWHM across the full *m*/*z* range. More accurately, the mean resolving power was calculated to be 3.12 × 10^6^ FWHM (3.07 × 10^6^ FWHM at *m*/*z* 400) and afforded the ability to resolve the very small mass differences required (see Table S5†). A total of 244 779 peaks were assigned molecular formulae with mass errors lower than 0.5 ppm, with an RMS mass error of 0.11 ppm for the whole mass spectrum (see Table 1). Mass splits for C_3_*vs.* S_1_H_4_ (3.37 mDa), C^13^C^14^N *vs.* H_3_O_3_ (1.79 mDa), C_4_*vs.*^13^CH_3_^32^S (1.10 mDa), and C^13^C^14^N *vs.* H_5_^34^S (0.56 mDa) have been reported as possibilities in previous works^37^ and were resolved across the full mass range. As a result of the current work, new, closely spaced mass splits were successfully resolved *e.g.* C_2_H_3_S *vs.*^13^CNO_2_ (0.71 mDa), N_2_O_2_H *vs.* C_4_^13^C (0.45 mDa), and ^13^C_2_NO_2_*vs.* C_6_ (0.39 mDa) (see details in Table S6†). To the best of our knowledge, this is the most complex fraction of petroleum that has been successfully characterized without the use of chromatography or MS/MS experiments, with a record in mass resolving power across the full *m*/*z* range, a record number of assignments of unique molecular formulae, and record range of DBE and carbon number.

**Table 1:** Figures of merit for the stitched mass spectrum of the ND-F sample

	Stitched
Average resolving power (*m*/*z* 260–1505)	3.12 × 10^6^
Resolving power at *m*/*z* 400	3.07 × 10^6^
Monoisotopic peaks assigned	106 871
Total peaks assigned	244 779
% Assigned	88.44%
RMS mass error for assigned peaks	0.11 ppm
Mean molecular weight	890.3 Da
Peaks with mass error ≤1 ppb	2305
Peaks with mass error ≤20 ppb	66 814
Peaks with mass error ≤50 ppb	122 911
Max. number of peaks assigned per Da	307

It is worth noting that the common mass difference of 3.37 mDa, corresponding to C_3_*vs.* S_1_H_4_, can span another peak within that same region, where a mass difference of 0.71 mDa correlates with C_2_H_3_S *vs.*^13^CNO_2_ (see Fig. 5). In order to obtain unambiguous assignments of the elemental composition of these species, a resolving power of 2 000 000 FWHM would be required at *m*/*z* 1600, for example. As shown in Fig. 6, this could potentially be achieved when adopting the OCULAR strategy described here. It is important, however, to take into account that a mass resolving power of 5 million FWHM at *m*/*z* 500 and 10 million FWHM at *m*/*z* 1000 would be required for an unambiguous assignment of even smaller mass differences within the composition of petroleum, such as 0.1 mDa.^79^

**Fig. 7:**
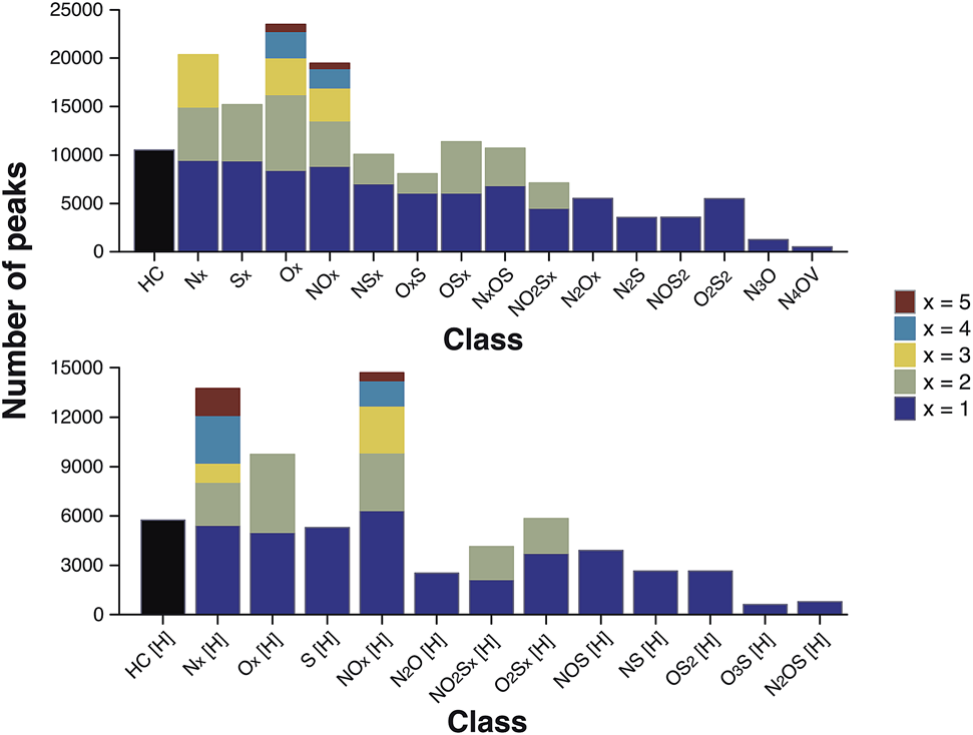
Number of peaks assigned per class for the ND-F sample. The compound classes are grouped by families. The number of peaks per class steadily decreases with increasing heteroatomic content. Compound classes with the label “[H]” (bottom) denote protonated species, which those classes without the label were observed as radical ions (above).

According to the “continuity model” developed by Boduszynski, the “compositional trends in fraction of increasing boiling points are continuous” and “this continuity extends even to nondistillable residues.”^73^ The compound class distribution of the stitched spectrum of the ND-F sample, grouped into class families, is shown in Fig. 7 and illustrates that species corresponding to the HC class (carbon and hydrogen only, no heteroatoms) were the most abundant, closely followed by species containing a single heteroatom (*x* = 1); also see alternative class distribution plots in Fig. S20 in the ESI.† As would be expected, there was a decrease in the total number of peaks assigned observed with respect to increasing heteroatom content.

**Fig. 8:**
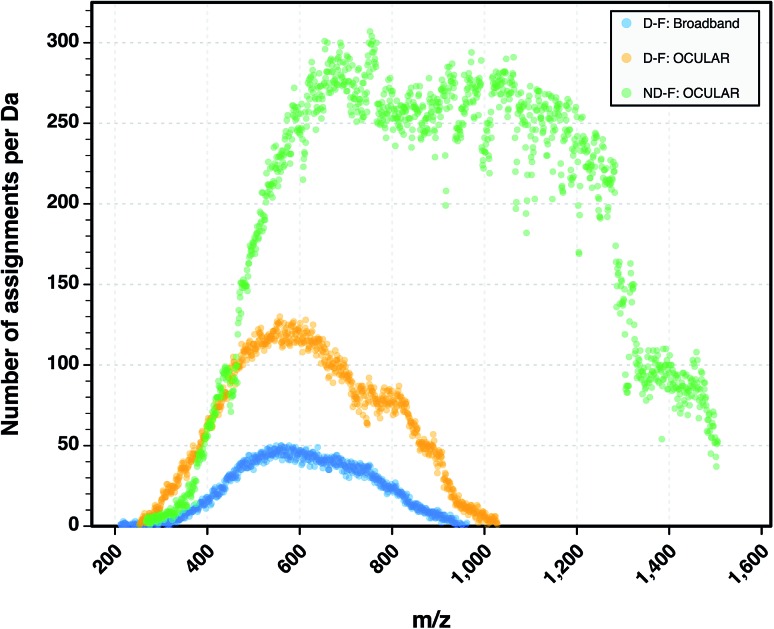
Comparison of the number of peaks assigned per nominal Da for the D-F mass spectra (both broadband and stitched) and the mass spectrum of the ND-F sample. A total of 244 779 molecular formulae were assigned using the OCULAR method for the ND-F sample, in contrast to 52 354 molecular formulae assigned for the D-F sample using the OCULAR method.

The heteroatomic composition is markedly more complex (61 distinct classes) for the maltenes of the ND-F compared with the species assigned in the D-F (40 distinct classes). The remarkable differences in complexity can be further seen in Fig. 8. The ND-F sample contained a factor of 4.67 more peaks assigned compared with equivalent data for the D-F sample. The number of assignments per dalton reaches its maximum above *m*/*z* 500. In line with the minimal number of ions needed for having a stable and detectable ion cloud (approximately 100 ions),^40^ a cell with a minimum capacity of 24.5 million ions would be required in order to detect all the components if they were of equal abundance; in practice, dynamic range requirements would dictate that the cell would need a significantly greater capacity. Thus, the extraordinary complexity, in terms of number of peaks and small mass differences, results in considerable space-charge effects in the collision cell and then the ICR cell, which prevent acquisition of a traditional broadband mass spectrum.

**Fig. 9:**
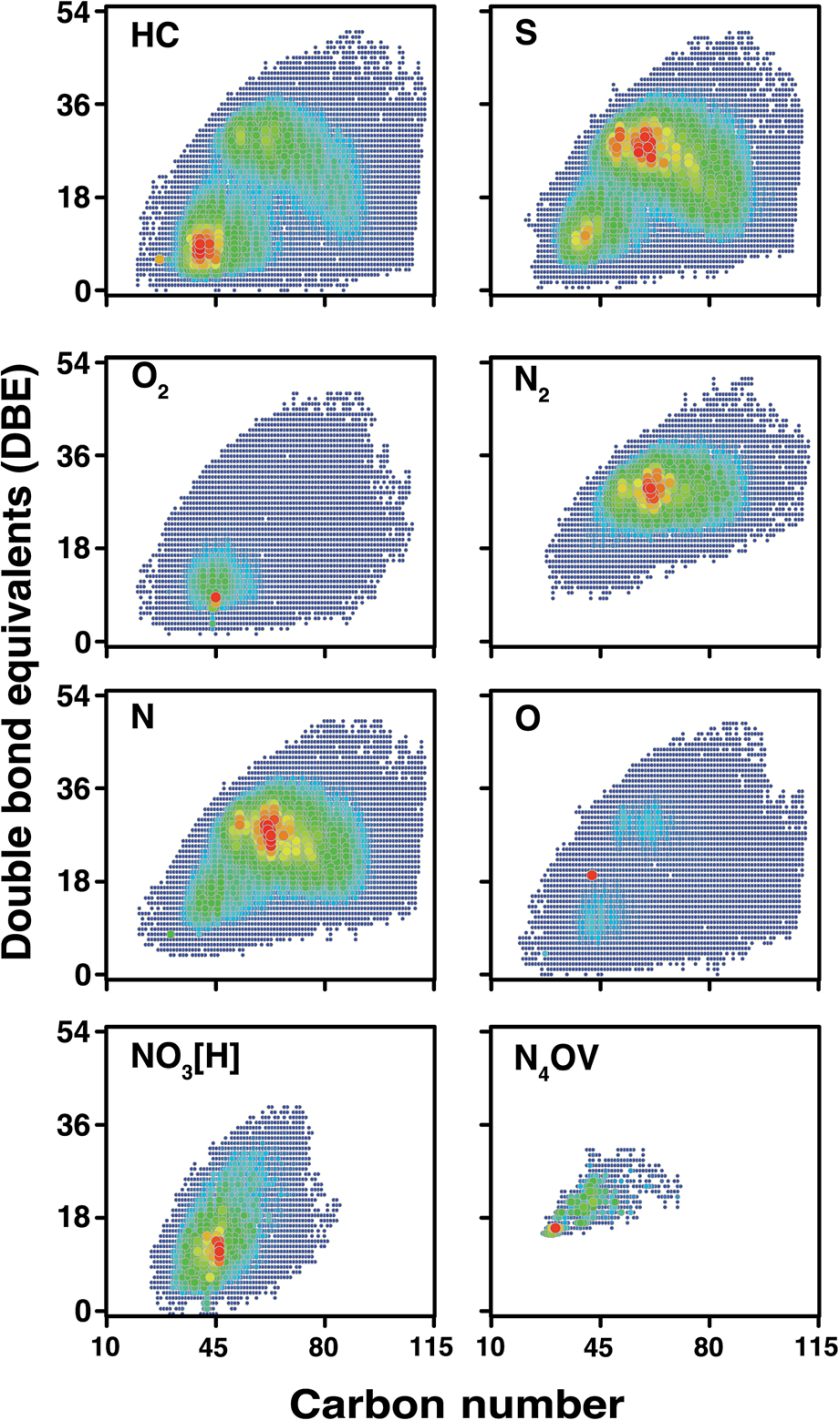
DBE *vs.* carbon number for selected compound classes detected in the stitched spectrum of ND-F sample. The colour scale indicates abundance, with blue indicating lowest abundances and red indicating highest abundances for each compound class. Petroleum components with DBE up to 51 have not been reported in literature previously.

According to the DBE *vs.* carbon number plots shown in Fig. 9, an unprecedented compositional space for a petroleum sample, spanning up to 51 DBE and 114 carbon atoms per molecule, is reported for the first time. It is worth noting that the sharp edges of the plots (rather than a more gradual change) at high carbon number for the HC, S, N, and O classes are indicative that further components will be present in the sample, completing the compositional space although at low abundance. This correlates with the observation of low-abundance signals spanning *m*/*z* 1500–1800, although mass spectra could not be acquired of viable quality for data analysis, due to the very low signal abundance. In contrast with other heteroatom classes, species of lower DBE (approximately DBE = 5–20) are predominant within the composition of the O_2_ class. A wide range of O_2_ class species were observed, however, using the OCULAR method; these include aliphatic species (DBE = 1), aromatic species, and highly polyaromatic forms (up to DBE = 47). Predominance of lower DBEs for the O_2_ class is consistent with previous work.^80^ Complete compositional space was found for compound classes even with such low relative abundances as 0.52% and 0.06% (NO_3_[H] and N_4_OV, respectively), as shown in Fig. 9. As seen in Table 1, approximately 50% of the compositions were assigned with a mass error ≤50 ppb and a mean distribution of the error close to 0.11 ppm was achieved throughout the full-range of the mass spectrum. An overview of the mass errors associated with the ND-F sample is represented by the violin plot shown in Fig. S21.†

### Future prospects and flexibility of OCULAR

The OCULAR method offers significant advantages in performance over traditional broadband experiments performed in FTMS instruments (including FT-ICR MS and Orbitrap). As can be seen in Table S2,† instruments with 7 T and 12 T magnets, for example, can achieve the same resolving power if longer acquisition times are achievable for the lower field instrument. However, if the signal decays quickly due to residual gas pressure, inharmonicity of electric and magnetic fields, and space-charge effects, using a longer acquisition time is not going to improve the resolving power. The introduction of the new dynamically harmonized cell^81^ (or “ParaCell”) in the new FT-ICR MS models such as the “solariX xR”, “solariX 2xR,” and “scimaX” instruments reduces the distortion of the ion cyclotron motion phases caused by non-ideal electric and magnetic fields, and thus, an increased lifetime of the time domain signal is possible. It is also possible to use quadrupolar (or 2*ω*) detection to increase the duty cycle or to increase the resolving power. For instance, Cho *et al.*^16^ demonstrated that it is possible to achieve a resolving power of 1.5 million at *m*/*z* 400 using quadrupolar detection with a 7 T solariX 2xR. This resolving power was compared to data acquired with higher magnetic field instruments using traditional detection; values of 1.2–1.5 million were cited for instruments with magnetic fields of up to 15 T. The OCULAR approach holds increasing promise for the future, therefore, as modern FT-ICR mass spectrometers with relatively low magnetic fields can operate more routinely with higher acquisition times.‡Online calculators for determining theoretical parameters: http://warwick.ac.uk/barrowgroup/calculators/fticr_tools/


As shown in Fig. 1, the number of segments, data set size and low *m*/*z* cut-offs can be varied according to specific needs. These can include: reduction of space-charge effects for complex samples, acquisition of data with sufficient resolving power to determine fine isotopic assignments, resolution of small mass splits, and lower mass errors associated with molecular assignments. Thus, the OCULAR method is highly flexible. For instance, an increased performance can be achieved even by the acquisition of only two segments, and the instrument time would take a total of approximately 30 min for a 4 M data set size. The highest levels of performance, however, can be achieved by acquiring narrow *m*/*z* segments and using larger data set size, which in turn, increases the total time required for a single stitched spectrum. In the case of the stitched spectrum for the D-F sample, produced using 41 4 M segments, a total of approximately 10 h of experiment time was necessary. Due to the extraordinary complexity of the ND-F sample, representing the largest number of unique assignments for a single sample, the highest levels of performance were required. Thus, for the ND-F sample, a larger data set size was used (8 M) to acquire a larger number of windows (65) to span the *m*/*z* range of this sample. Although the acquisition of the data for the ND-F sample required approximately 48 h, it should be emphasized that this sample represents an extreme case due to its complexity, where traditional methods for characterization had previously failed. For simpler samples, which are nonetheless considered complex, wider segments with lower data set size can be acquired. For instance, a bio-oil sample has been characterized using the range *m*/*z* 220–735, acquired by segmenting the data using windows of 4 M data set size and with an *m*/*z* width of 30; this experiment required 3.18 h using a 12 T solariX instrument (data not shown).

## Conclusions

An unprecedented and near constant ultrahigh resolving power of approximately 3 million FWHM across a broad *m*/*z* range (*m*/*z* 260–1500) has been demonstrated using the OCULAR method, expanding the *m*/*z* range of resolved peaks, increasing the number of molecular formula assignments, and increasing the confidence in the assignments. A record number of 244 779 unique molecular formulae have been assigned for a single sample; this was achieved without the aid of dissociation (MS/MS) experiments, which would increase numbers of peaks as a result of fragmentation, or the use of chromatography. Furthermore, this data quality was obtained using a 12 T FT-ICR MS instead of the very highest field (15 T or 21 T); even greater improvements may be expected when using the very highest magnetic fields available. The mass errors associated with the assignments were approximately halved, compared to traditional methods; approximately 98% of the detected species could be assigned within an RMS mass error of 0.10 ppm. The OCULAR method has been used to produce mass spectra segments spanning *m*/*z* widths of approximately 20, the segments have been acquired using increasing acquisition time with *m*/*z* in order to maintain constant resolving power across the *m*/*z* range (unlike traditional FT-ICR MS experiments), and a novel algorithm named Rhapso has been developed to automatically trim the segments, compensate for signal attenuation at the edges of each isolation range, determine appropriate regions of overlap, and stitch the segments together to produce the final mass spectrum. The resulting data offers improved dynamic range, resolving power, and mass accuracy, with increased numbers of molecular formula assignments and the ability to monitor petroleum compositional space over a broader DBE range and carbon number range than previously achieved. Due to the data quality, assignments are obtained with higher mass accuracy but can also be confirmed by the monitoring of well-resolved isotopic patterns over the full mass range of detection. Thus, the ultrahigh performance of FT-ICR mass spectrometers can be maintained across the whole mass spectrum, instead of being optimal at the lower *m*/*z* end of a given mass range. The strategy described in this work was tested in the analysis of the D-F sample and truly ND-F sample of heavy South American petroleum, the latter a sample exhibiting extraordinary complexity. The OCULAR method advances the capabilities of modern FT-ICR instruments (whether high or low magnetic field, commercially available or home-built), significantly improving data quality and facilitating detailed characterization of samples considered too challenging for conventional broadband experiments. As an application to one of the greatest analytical challenges, the OCULAR method has been demonstrated for heavy petroleum samples. The method can equally be applied to other applications requiring the highest levels of performance from Fourier transform mass spectrometers, such as FT-ICR and Orbitrap instruments, both enhancing current capabilities of lower performance instruments and pushing the current analytical limits when using state-of-the-art instrumentation.

## Conflicts of interest

There are no conflicts to declare.

## Supplementary Material

Supplementary information
