# Supplementary material for: Pushing the analytical limits: new insights into complex mixtures using mass spectra segments of constant ultrahigh resolving power
Source: Chem Sci. 2019 Jul 5;10(29):6966–78. doi: 10.1039/c9sc02903f (PMC6764280; doi:10.1039/c9sc02903f)
Supplement: Supplementary file 1 [file SC-010-C9SC02903F-s001.pdf]

Electronic Supplementary Material (ESI) for Chemical Science.
This journal is © The Royal Society of Chemistry 2019

Supplementary Information

Pushing the Analytical Limits: New Insights into Complex Mixtures using Mass Spectra Segments of Constant Ultrahigh Resolving Power

Diana Catalina Palacio Lozano,[a,b] Remy Gavard,[c] Juan P. Arenas-Diaz,[b] Mary J. Thomas,[a,c] David Stranz,[d] Enrique Mejía-Ospino,[b] Alexander Guzman,[e] Simon E. F. Spencer,[f] David Rossell,[g] Mark P. Barrow[a*]

a. Department of Chemistry, University of Warwick, Coventry, CV4 7AL, United Kingdom

b. Department of Chemistry, Universidad Industrial de Santander, Bucaramanga, Colombia

c. Molecular Analytical Science Centre of Doctoral Training, University of Warwick, Coventry, CV4 7AL, United Kingdom

d. Sierra Analytics Inc., Modesto, California, USA

e. Instituto Colombiano del Petróleo, Piedecuesta, Ecopetrol, Colombia

f. Department of Statistics, University of Warwick, Coventry, CV4 7AL, United Kingdom

g. Department of Economics & Business, Universitat Pompeu Fabra, Barcelona 08005, Spain

Table of Contents

1. Sample details 2

2. OCULAR method 4

3. Distillable sample: D-F 12

4. Space-charge effects in extremely complex samples 20

5. Truly non-distillable sample (ND-F) 21

References 28

1. Sample details

Elemental analysis of the distillable fraction, non-distillable fraction, and the vacuum residue (D-F, ND-F, and VR, respectively) can be found in Table S1. According to the API gravity (American Petroleum Institute), the fractions D-F and ND-F can be classified as heavy and extra-heavy, respectively.

Distillable fraction. A sub-fraction of a South American vacuum residue was obtained by supercritical fluid extraction (SFEF). The supercritical solvent was n-hexane and the conditions were a temperature of 265 °C and pressure of 4.5 MPa. The SFEF process has been described elsewhere.[1] As seen in Fig. S1, 90% of the constituents of these fractions have boiling points lower than 720 °C atmospheric equivalent temperature (AET).

Truly non-distillable fraction. A short path distillation unit operating at 687 °C AET was used to obtain a truly non-distillable fraction. According to the true boiling point curve (Fig. S1), only 18% of its constituents evaporated below 720 °C AET. Thus, 82% of the constituents

had boiling points with an unknown final boiling point; the heaviest components may not be vaporizable.[2] Considering the extraordinarily high complexity of this sample, the sample was fractionated by solubility in n-heptane (maltenes), which corresponded to 89% of the sample (51% resins, 33% aromatics, and 1% saturates). The maltene fraction was removed from the non-distillable sample by Soxhlet extraction using n-heptane for a period of 12 h.

Table S1
Elemental analysis of the South American vacuum residue, distillable fraction, and non-distillable fraction. Density was measured by means of a digital density meter (ASTM D4052),[3] SARA composition analysis by ASTM D2007,[4] and sulfur percentage by ASTM D1552.[5]

	VR	DF	ND-F
S (Wt. %)	1.8	1.45	1.89
Density	1.0251	0.9766	1.0404
API°	6.5	12.9	4.5
Saturates	12	35.4	1
Aromatics	46	50.7	33
Resins	37	13.1	51
Asphaltenes	4.9	0.7	14.6

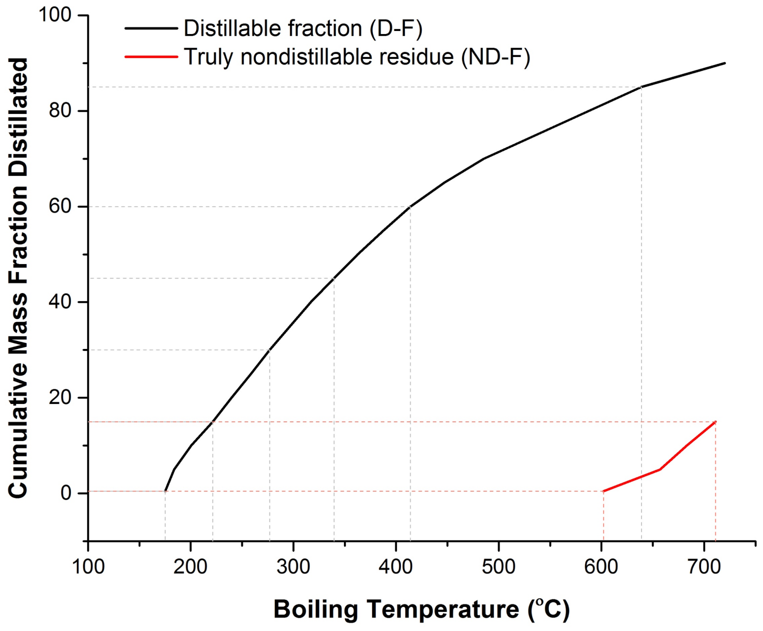

Fig. S1 True boiling point curves of the analysed samples. The boiling temperatures were obtained by high temperature simulate distillation ASTM 7169 that can characterise the oil distillation temperature up to 740 °C AET.[6]

2. OCULAR method

The mass resolving power in FT-ICR MS can be estimated by Equation 1

$$R \propto \frac{qBT_{acq}}{mk} \qquad (1)$$

where R is the resolving power, q, B, m, k and T_{acq} are the charge of the ion, strength of magnetic field, mass of the ion, peak width constant, and length of the time domain data (acquisition time), respectively.[7] Therefore, the resolution can be improved by recording the time domain signal for a longer durations (larger T_{acq}). The length of the time domain signal, T_{acq} , is also influenced by physical factors such as background gas pressure, inharmonicity of electrostatic field in the cell, space-charge effects, and magnetic field inhomogeneity, among others.[8] Thus, for optimum resolution, the acquisition time should be as long as possible and

the time domain signal should last the full duration. The acquisition time can be calculated using the Equation 2

$$T_{acq} = \frac{N}{f_s} \qquad (2)$$

where N and f_s are experimental parameters: the data set size and the sampling frequency, respectively. In accordance with the Nyquist theory, the sampling frequency must be twice the frequency of the lowest m/z being detected; hence, it determined by the low m/z cut-off values of the mass spectra.[9]

Under ideal experimental conditions (e.g. no collisions with residual gas, ideal electric and magnetic fields, and no space-charge effects), FT-ICR mass spectrometers of lower magnetic field can reach the same resolving power as higher field instrumentation if the duration of the time domain signal is sufficiently high to compensate for the lower magnetic field (see Equation (1) and Table S2). For instance, a resolution of approximately one million (FWHM) can be obtained at *m/z* 850 using a 7 T FT-ICR with an acquisition time of 10.388 s at 4M, while the same resolving power could be obtained using a 12 T instrument if the transient lasts 6.060 s. It can be challenging, however, to produce longer transients, particularly due to space-charge effects, and so higher field instrumentation offers an advantage.

Table S2
Examples of the acquisition times required for a target, constant resolving power; three selected segments with different low *m/z* cut-offs, two magnetic fields, and two data set sizes are shown. The resolving powers afforded using the traditional broadband method are provided for comparison.

	Magnetic field strength (T)	Low *m/z* cut-off*	*m/z* for measured resolving power	Resolving power (x10^6)		Acquisition time (s)	
				4 M	8 M	4 M	8 M
OCULAR	12	120	200	1.070	2.138	1.398	2.797
	7	120	200	1.070	2.138	2.397	4.794
	12	520	850	1.090	2.180	6.060	12.119
	7	520	850	1.090	2.180	10.388	20.775
	12	857	1400	1.090	2.181	9.987	19.973
	7	857	1400	1.090	2.181	17.120	34.240
Broadband	12	120	850	0.515	0.503	1.398	2.797
	7	120	850	0.515	0.503	2.397	4.794

* The required low *m/z* cut-off can be calculated, such as by using available web resources (e.g. warwick.ac.uk/barrowgroup/calculators/fticr_tools/)

According to Equations (1) and (2), the resolving power can be increased by increasing the low *m/z* cut-off for detection. For instance, as can be seen in Fig. S2, the resolving power at 600 Da for a mass spectrum increases 2x and 3x when the low *m/z* cut-off is increased from m/z 200 to *m/z* 400 and *m/z* 600 Da, respectively. Thus, an improvement in the resolving power of a broadband mass spectrum can be achieved by segmenting the mass range into narrower windows (see Fig. S2). It is important to notice that the resolving power still decreases with *m/z* per segment and thus use of narrower segments offers greater improvement over the traditional broadband experiment. As can seen in Fig. S2, the acquisition time of the consecutive windows must be increased to maintain the same resolving power as observed for the lowest *m/z* detected; in this way, near constant resolving power can be achieved by use of narrow *m/z* windows.

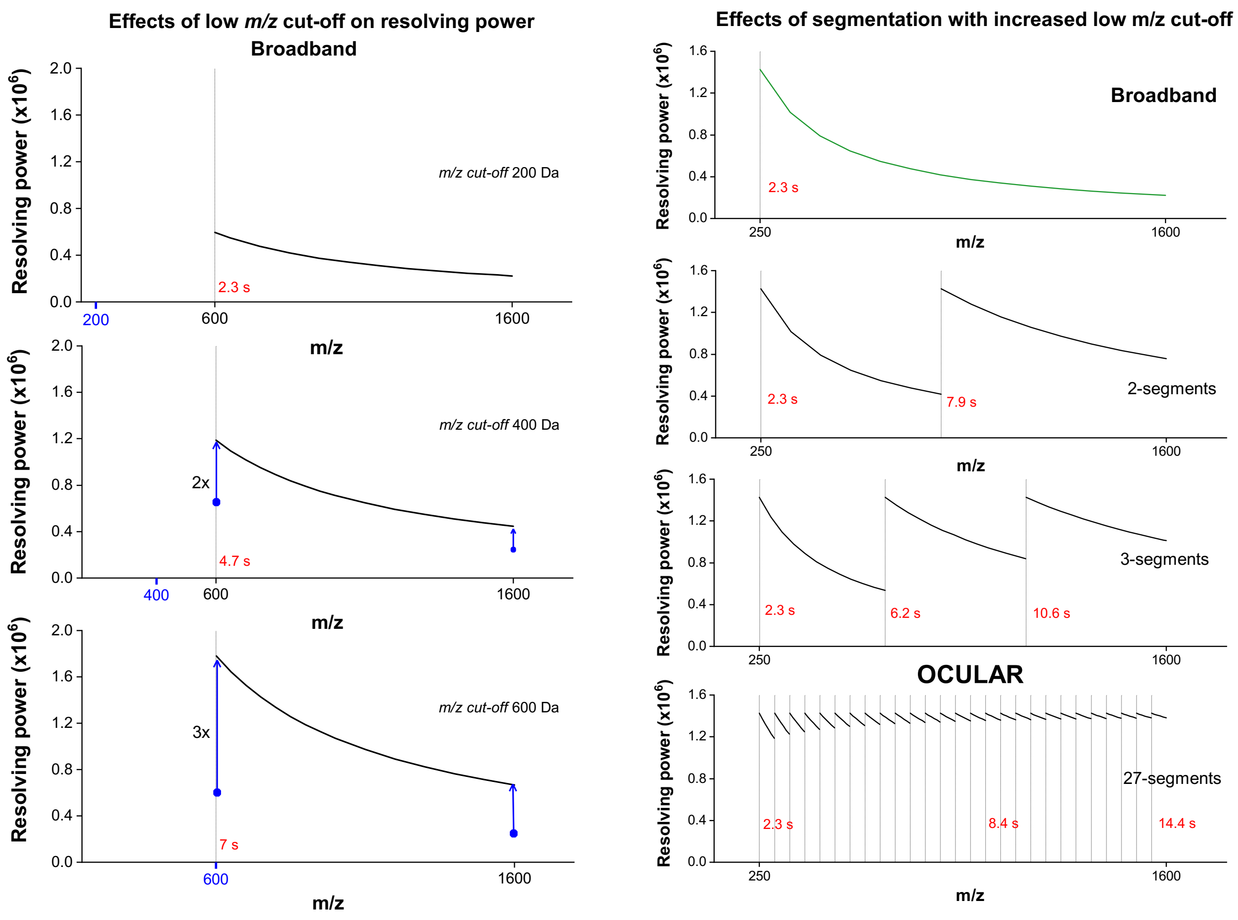

Fig. S2 Left: Resolving power for broadband mass spectra within a range of *m/z* 600-1600, using low *m/z* cut-off at *m/z* 200, *m/z* 400, *m/z* 600. Raising the low *m/z* cut-off (values shown in blue) increases the acquisition time and therefore the overall resolving power. Right: Comparison of the resolving power of a broadband spectrum acquired between *m/z* 250-1600 with segmented mass spectra with sequentially increased acquisition time. Near constant resolution can be achieved through use of smaller data segments. The numbers shown in red correspond to the acquisition times. To simplify the OCULAR figure, the acquisition times are only shown for the first, middle, and last segments.

The OCULAR method includes the use of a quadrupole to isolate *m/z* ranges of interest, For sequential *m/z* segments, the acquisition times are increased to result in near constant resolving power across the entire *m/z* range (multiple segments). Fig. S3 shows the acquisition times (T_{acq}) for each segment for the D-F and ND-F samples. For the D-F sample, 4 M data set sizes were used and 41 segments were acquired; for the ND-F sample, 8 M data set sizes were used and 65 segments were acquired.

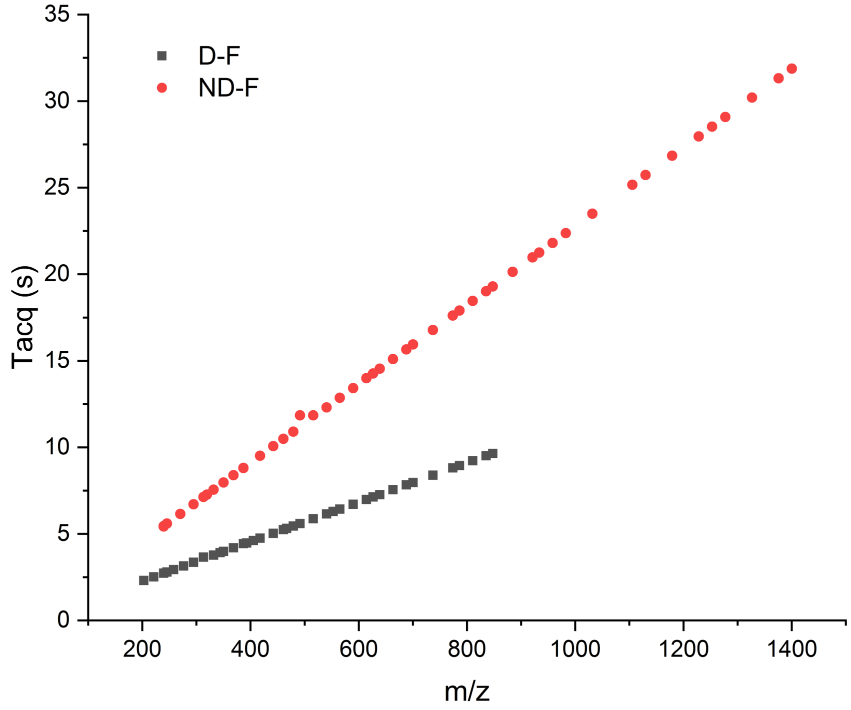

Fig. S3 Acquisition time per segment for the OCULAR experiment, applied to a distillable fraction (D-F) and a truly non-distillable fraction (ND-F) of a petroleum sample.

The low *m/z* cut-off and sampling frequency per segment of the stitched spectra are reported in Table S3 for both the D-F and ND-F samples.

Table S3
Low *m/z* cut-off, frequency, and acquisition time (T_{acq}) per window of the data for the ND-F and D-F samples

Segment center	ND-F (8 M data sets)					D-F (4 M data sets)				
	#	Low *m/z* cut-off	T_{acq}	Highest frequency (kHz)	Sampling frequency, 2f (kHz)	#	Low *m/z* cut-off	T_{acq}	Highest frequency (kHz)	Sampling frequency, 2f (kHz)
260						1	202.6912	2.3069	909.0910	1818.1820
278	1	239.5439	5.4260	769.2310	1538.4620	2	221.1175	2.5166	833.3330	1666.6660
295	2	239.5439	5.4526	769.2308	1538.4616	3	239.5439	2.7263	769.2310	1538.4620
312	3	245.6860	5.5924	750.0001	1500.0002	4	245.6860	2.7962	750.0010	1500.0020
330	4	270.2544	6.1516	681.8181	1363.6362	5	257.9702	2.9360	714.2800	1428.5600
348	5	294.8228	6.7109	625.0000	1250.0000	6	276.3965	3.1457	666.6670	1333.3340
366	6	313.2491	7.1303	588.2352	1176.4704	7	294.8228	3.3554	625.0001	1250.0002
384	7	319.3911	7.2701	576.9230	1153.8460	8	313.2491	3.6551	588.2300	1176.4600

402	8	331.6753	7.5498	555.5553	1111.1106	9	331.6753	3.7750	555.5550	1111.1100
420	9	350.1015	7.9692	526.3157	1052.6314	10	343.9595	3.9147	535.7150	1071.4300
438	10	368.5277	8.3886	500.0010	1000.0020	11	350.1015	3.9846	526.3160	1052.6320
456	11	386.9539	8.8037	476.1902	952.3804	12	368.5277	4.1943	500.0010	1000.0020
475	12	386.9539	8.8084	476.1905	952.3810	13	386.9539	4.4404	476.1903	952.3806
494	13	417.6642	9.5071	441.1765	882.3529	14	393.0960	4.4740	468.7501	937.5002
513	14	442.2324	10.0663	416.6666	833.3332	15	405.3801	4.6137	454.5460	909.0920
531	15	442.2324	10.0663	416.6666	833.3332	16	417.6642	4.7535	441.1765	882.3530
550	16	460.6585	10.4858	400.0001	800.0002	17	442.2324	5.0332	416.6670	833.3340
568	17	479.0846	10.9052	384.6153	769.2306	18	460.6585	5.2488	400.0010	800.0020
587	18	491.3687	11.8491	375.0001	750.0002	19	466.8005	5.3128	394.7370	789.4740
605	19	515.9367	11.8491	357.1429	714.2858	20	479.0846	5.4526	384.6152	769.2304
624	20	540.5048	12.3033	340.9091	681.8182	21	491.3687	5.5924	375.0010	750.0020
643	21	540.5048	12.3033	340.9091	681.8182	22	515.9367	5.8702	357.1429	714.2858
662	22	565.0728	12.8625	326.0869	652.1738	23	540.5048	6.1565	340.9090	681.8180
681	23	589.6408	13.4218	313.0001	626.0002	24	552.7888	6.2915	333.3330	666.6660
700	24	614.2088	13.9902	300.0010	600.0020	25	565.0728	6.4313	326.0869	652.1738
718	25	626.4927	14.2606	294.1175	588.2350	26	589.6408	6.7109	312.5001	625.0002
737	26	638.7767	14.5406	286.4615	572.9230	27	589.6408	6.7109	312.5001	625.0002
756	27	638.7767	14.5405	286.4615	572.9230	28	614.2088	6.9905	300.0010	600.0020
775	28	663.3446	15.0995	277.7778	555.5556	29	626.4927	7.1302	294.1177	588.2353
794	29	663.3446	15.0995	277.7778	555.5556	30	638.7767	7.2701	289.4615	578.9230
813	30	687.9124	15.6587	267.8572	535.7144	31	663.3446	7.5498	277.7770	555.5540
832	31	700.1963	15.9384	263.1579	526.3158	32	663.3446	7.5498	277.7770	555.5540
851	32	737.0480	16.7771	250.0001	500.0002	33	687.9124	7.8294	267.8571	535.7142
870	33	773.8996	17.6161	238.0952	476.1905	34	700.1963	7.9692	263.1590	526.3180
889	34	786.1835	17.8957	234.3750	468.7500	35	737.0480	8.3886	250.0010	500.0020
908	35	810.7512	18.4549	227.2727	454.5454	36	737.0480	8.3886	250.0010	500.0020
927	36	835.3188	19.0142	220.5883	441.1765	37	773.8996	8.8080	238.0952	476.1904
946	37	835.3188	19.0142	220.5883	441.1765	38	786.1835	8.9478	234.3750	468.7500
965	38	847.6026	19.2937	217.3913	434.7826	39	810.7512	9.2276	227.2729	454.5458
984	39	847.6026	19.2937	217.3913	434.7826	40	835.3188	9.5071	220.5882	441.1765
1002	40	847.6026	19.2937	217.3913	434.7826	41	847.6026	9.6469	217.3913	434.7826
1020	41	884.4540	20.1327	209.3333	418.6667					
1038	42	884.4540	20.1327	209.3333	418.6667					
1056	43	921.3053	20.9715	200.0001	400.0002					
1074	44	921.3053	20.9715	200.0001	400.0002					
1093	45	933.5891	21.2512	197.3684	394.7368					
1113	46	933.5891	21.2512	197.3684	394.7368					
1133	47	958.1566	21.8104	192.3077	384.6154					
1153	48	982.7240	22.3696	187.5001	375.0002					
1173	49	982.7240	22.3696	187.5001	375.0002					
1193	50	1031.8588	23.4981	178.5760	357.1520					
1213	51	1105.5608	25.1658	166.6666	333.3332					
1233	52	1105.5608	25.1658	166.6666	333.3332					
1253	53	1130.1280	25.7281	163.0400	326.0800					
1273	54	1179.2624	26.8436	156.2501	312.5002					
1293	55	1179.2624	26.8436	156.2501	312.5002					

1313	56	1179.2624	26.8436	156.2501	312.5002
1333	57	1228.3967	27.9622	150.0001	300.0002
1353	58	1252.9638	28.5213	147.0590	294.1180
1373	59	1277.5308	29.0805	144.2401	288.4802
1393	60	1326.6648	30.1999	138.8880	277.7760
1413	61	1326.6648	30.1999	133.9280	267.8560
1433	62	1326.6648	30.1999	133.9280	267.8560
1453	63	1375.7987	31.3175	133.9280	267.8560
1473	64	1375.7987	31.3175	133.9286	267.8572
1493	65	1400.3656	31.8767	131.5789	263.1579

Table S4
Acquisition parameters of the spectra in broadband mode and using the OCULAR method

	**Broadband D-F**	**OCULAR D-F**	**OCULAR ND-F**
Flow rate		500 μL/h	
Drying gas temperature		250 °C	
Vaporizer temperature		350 °C	
Capillary voltage		1200 V	
Excitation setting		20 %	
Trapping: back plate		0.4 V	
Trapping: front plate		0.4 V	
Concentration	0.05 mg/mL	0.05 mg/mL	0.04 mg/mL
Data size	4 MW	4 MW	8 MW
Range of acquisition time	3.2 s	2.3-10.7 s	5.4-32 s
Number of segments acquired	1	41	65
Accumulated scans	100	50/segment	100/segment
Ion accumulation time	0.03 s	0.6 s	5.0 s
m/z range or width	2750	24	24
Low mass cut-off for detection	*m/z* 200	Increased in-line with segments	Increased in-line with segments
Low mass cut-off for excitation	250	250	250
Spectrum mode	Absorption	Absorption	Absorption

2.1 Rhapso

A flowchart of the steps required to stitch the data using the in-house software “Rhapso” can be seen in Fig. 1 in the main text. An example of the result of step 1 is illustrated in Fig. S4, with a before and after trimming of the segment between *m/z* 557-580. After further steps, Fig. S5 shows the intensity correction for the segment *m/z* 595-616; mass spectra resulting from traditional broadband acquisition, stitching without intensity correction, and the OCULAR method could then be compared.

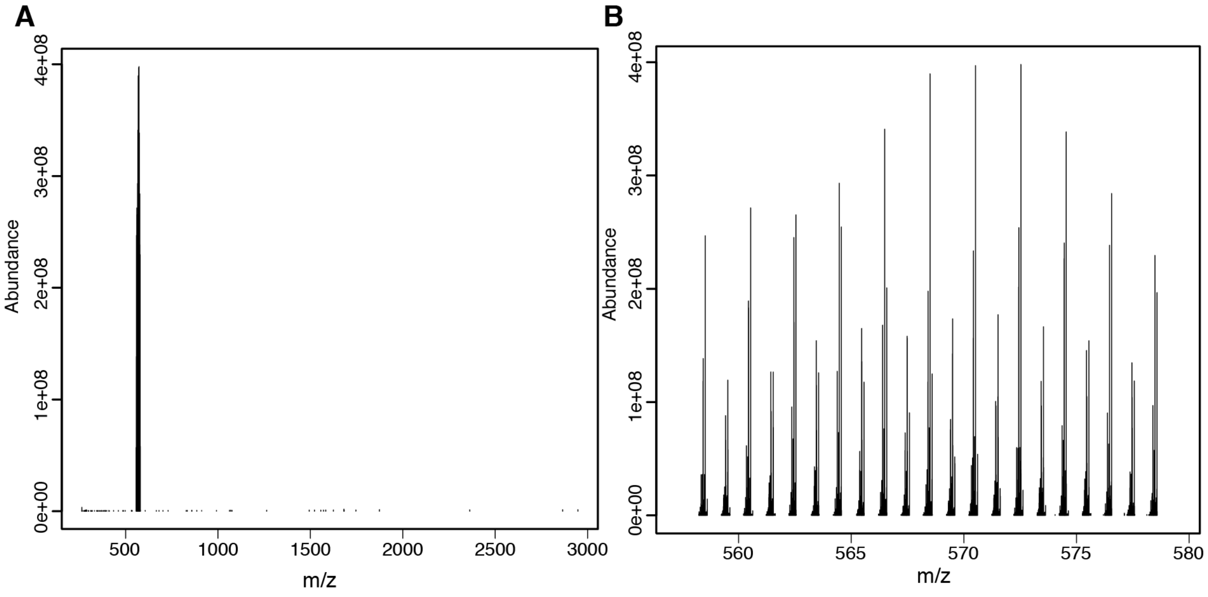

Fig. S4 Plot of the spectrum (A) before trimming for the targeted mass range *m/z* 558-579 and (B) after automatic noise removal

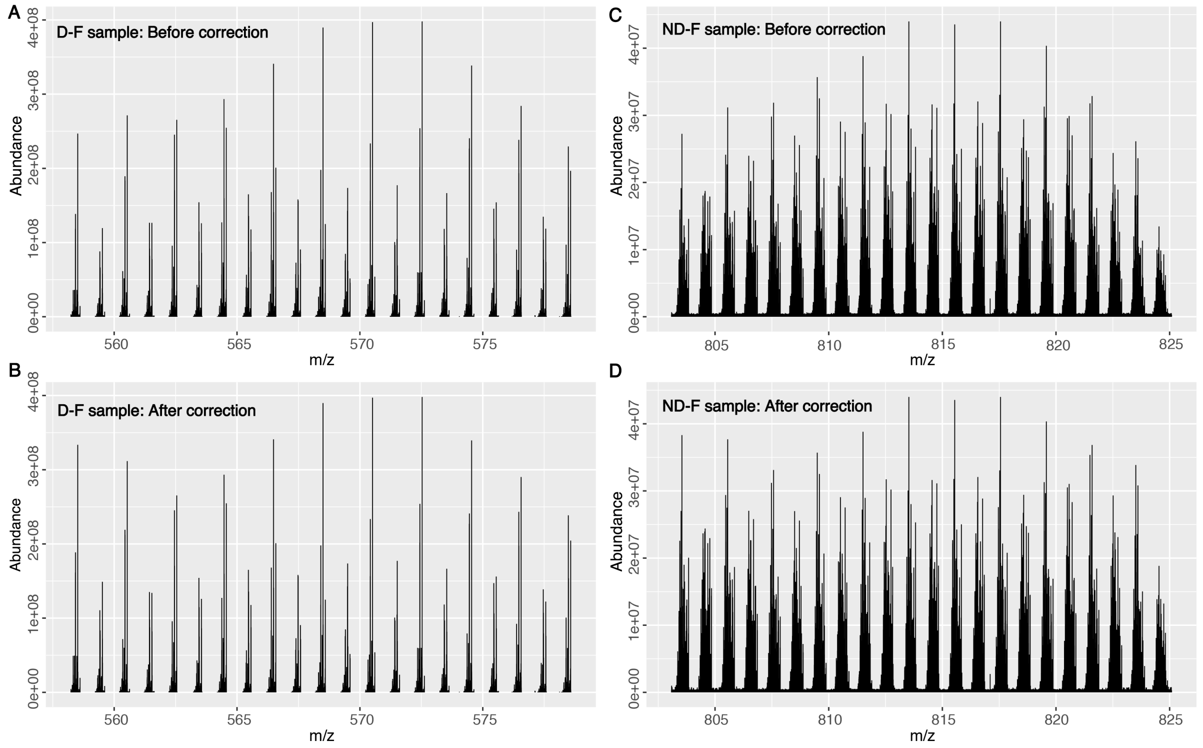

Fig. S5 Segments for the D-F (A and B, left) and ND-F samples (C and D, right), both before (top) and after (bottom) intensity correction. The natural undulations are preserved while correcting the signal attenuation due to the isolation process using the quadrupole.

3. Distillable sample: D-F

As a result of the ultrahigh resolution of the stitched spectra and the improved dynamic range, the number of peaks assigned were increased by three-fold compared with the broadband absorption mode mass spectrum (4.6-fold compared with the broadband magnitude mode mass spectrum, see Fig. S6). The assigned peaks were plotted as contributions by heteroatomic class, $C_cH_hN_nO_oS_s$, (Fig. S7). Compound classes with the label "[H]" denote protonated species, which those classes without the label were observed as radical ions. According to this Figure, approximately twice as many radical ions were observed in the stitched mass spectrum and approximately twice as many compound classes became accessible e.g. OS_2, O_3, N_2O, N_2, N_4OV (see Fig. S7, among others). It is worth noting that the relative contributions of radical ions versus protonated species are influenced by experimental parameters, such as sample

infusion rate.[10] The total number of peaks assigned were significantly increased for each class, although the relative abundance (in percentage) of the classes remained similar.

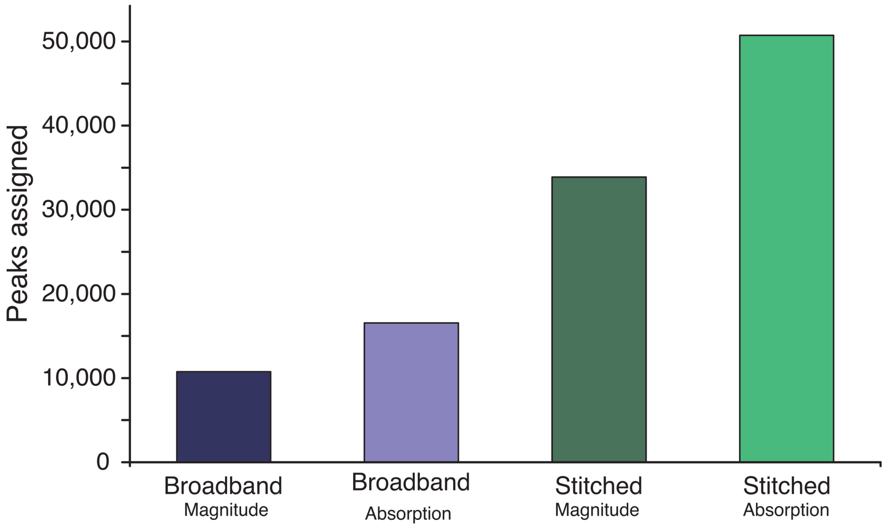

Fig. S6 Number of molecular compositions assigned for the D-F mass spectra obtained using different methods: magnitude mode vs. absorption mode, and broadband vs. stitching.

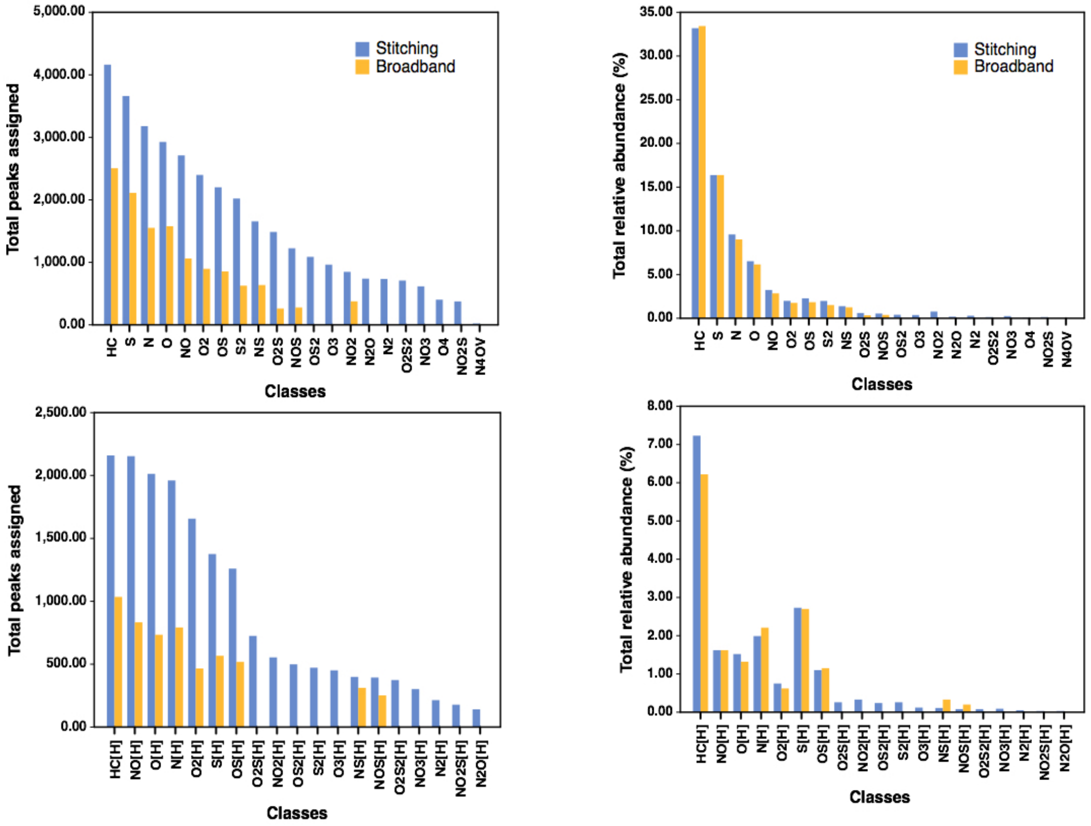

Fig. S7 Class distribution comparing the number of peaks and total relative abundance of the assignments obtained for the broadband and OCULAR data for the D-F sample.

As can be seen in Fig. S8 and Fig. S9, more peaks were assigned at the extremities of the DBE and carbon number ranges for each class in the OCULAR data. Similarly, homologous series of species in low percentage of abundance (e.g. N_2O at 0.18%) were clearly assigned in the compositional space defined by the DBE and carbon number. Thus, the ultrahigh mass resolution and mass accuracy attained using the OCULAR method affords new abilities to reveal the compositional space of species in lower abundance. In agreement with previous findings,[11,12] the combination of narrow windows increases the number of ions assigned as a result of the enhanced S/N at the low and high mass ends of the mass distribution and the improved dynamic range when more ions can be accumulated for longer periods of time with reduced space-charge effects.

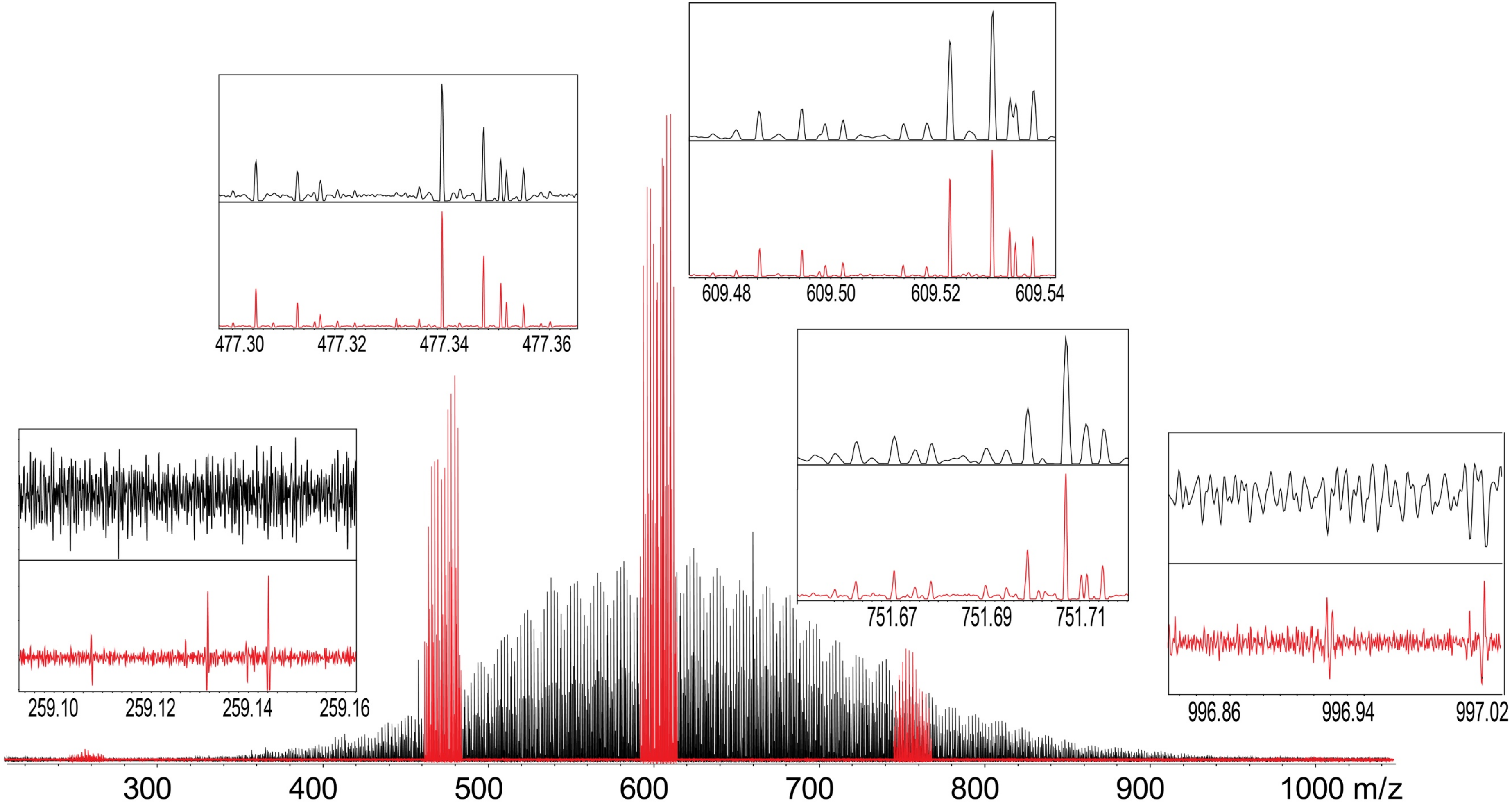

Fig. S8 In black, mass spectrum of the distillate sample in broadband mode. In red, five mass spectra acquired using isolation windows for narrow ranges as examples to demonstrate performance improvements. The inserts show enlarged *m/z* ranges of the data acquired using both methods. All data are shown in absorption mode

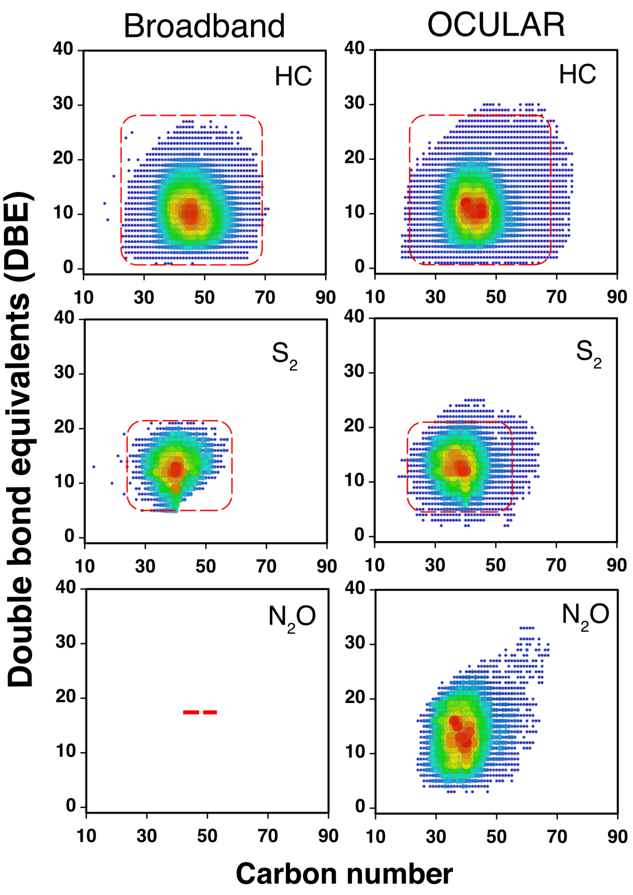

Fig. S9 Plots of DBE versus carbon number of selected classes in broadband mode (left) and using the OCULAR method (right) for the D-F sample. The N_2O class was not detected at all in the broadband spectrum.

The mean resolving power per narrow window is almost constant (average of 1,792,000 FWHM) over the full mass range (see Fig. S10). As shown in Fig. S11 and Table S5, about the half of the assigned peaks correspond to the isotopologues that were resolved and assigned with low ppm error (<0.5 ppm) in the mass spectrum, even at higher masses (Fig. S12). Thus, the isotopic contributions from $^{13}C4$, $^{34}S2$, and $^{13}C^{34}S$ assist in the correct assignment of molecular formulae.

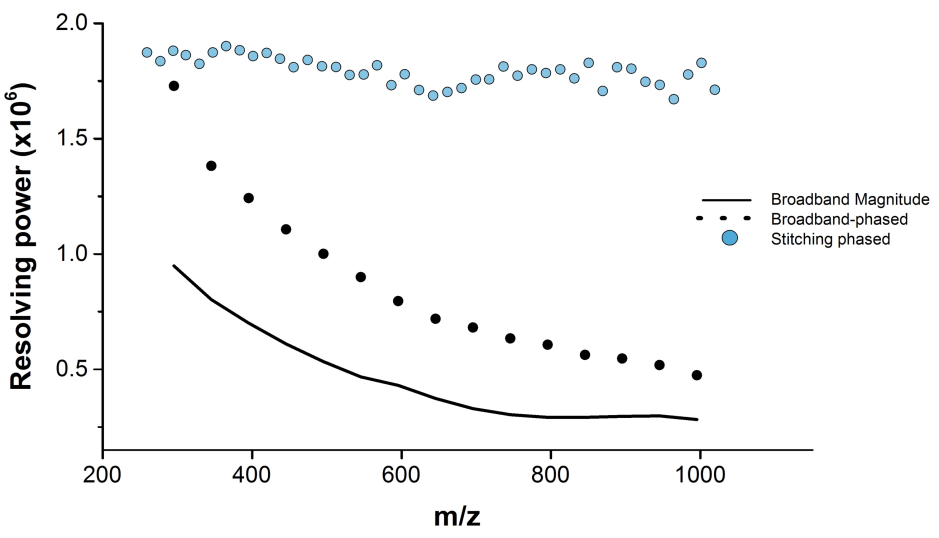

Fig. S10 Comparison of the average resolving power as a function of *m/z* for the D-F sample using: a broadband mass spectrum using magnitude mode, a broadband mass spectrum using absorption mode ("phased"), and a corrected stitched data set using absorption mode (the OCULAR method), using the mean resolution of each segment.

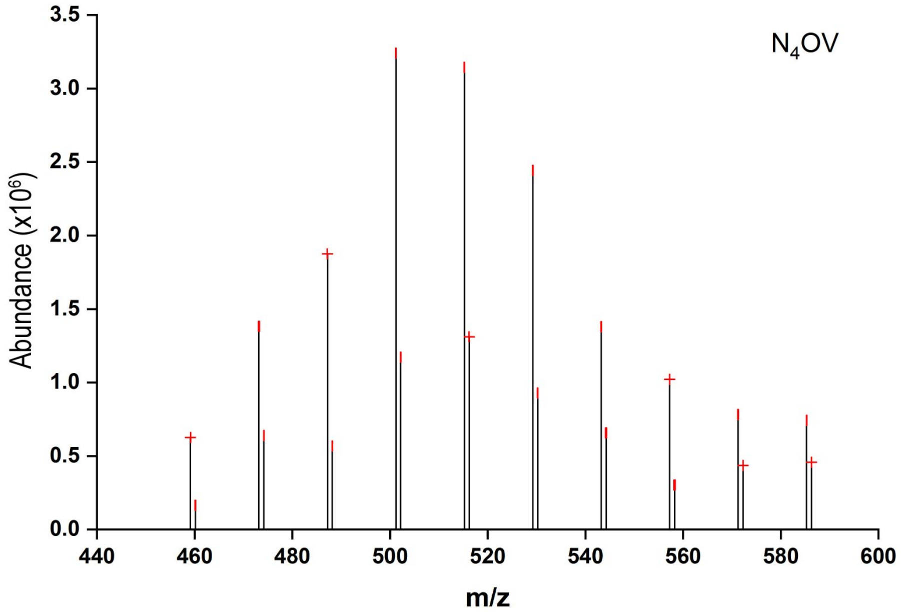

Fig. S11 Mass distribution of a homologous series (17 DBE) for the N_4OV class found in the OCULAR data for the stitched spectrum of the D-F sample, with a relative abundance of only 0.01% (see Figure S8). The confidence in the assignment is based on the low mass errors (horizontal bars in red) and the isotopic distribution.

Table S5

Isotopic number of peaks detected in broadband and OCULAR method for the D-F sample (not detected denoted by "-").

Isotope	OCULAR	Broadband
Monoisotopic	24,878	10,585
$^{13}C_1$	15,549	5,238
$^{13}C_2$	8,572	1,824
$^{13}C_3$	1,115	219
$^{13}C_4$	64	-
$^{34}S_1$	591	348
$^{13}C_1{}^{34}S_1$	420	182
$^{13}C_2{}^{34}S_1$	52	3
$^{18}O_1$	2	-
$^{15}N_1$	119	-

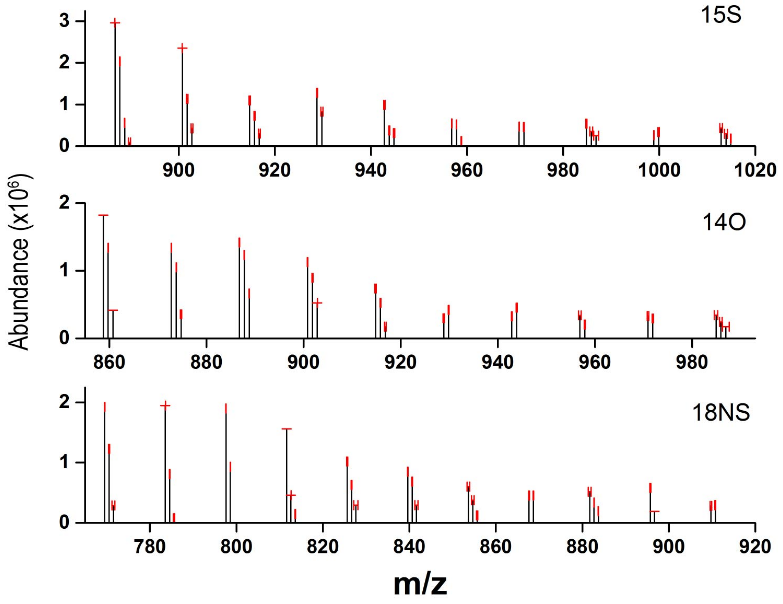

Fig. S12 Isotopic contributions at the tail of selected homologous series, observed at low S/N within the D-F sample data. Bars in red indicate the mass error of the assignment, in ppm. DBE values are indicated by the number preceding the class name (e.g. 18 NS corresponds to a class NS with a DBE of 18)

To illustrate that ultrahigh resolution is also necessary to improve mass accuracy, a comparison of a zoom-in of the mass distribution is shown in Fig. S13. At *m/z* 637, a resolution of 519,000 FWHM is not enough to baseline resolve and assign molecular compositions to peaks with a mass difference of 1.10 mDa, corresponding to a difference of $^{12}C_4$ vs $^{13}CH_3S$. At higher masses, such as *m/z* 793, peaks with this same mass difference are no longer resolved and coalescence of the peaks is observed. In this particular case, both assignments are possible within the tolerable mass error. Only after attaining the ultrahigh resolution achieved in the OCULAR data could it be demonstrated that both assignments were valid.

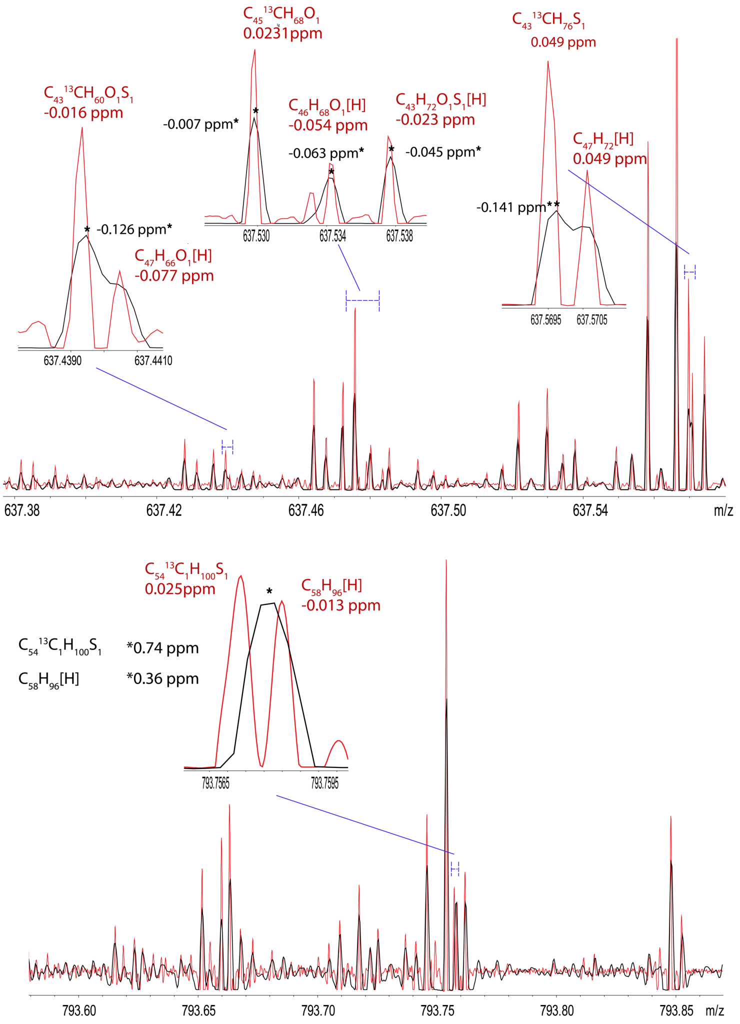

Fig. S13 Comparison of enlarged regions for *m/z* 637 and *m/z* 793, with data acquired using broadband mode (dark line) and the OCULAR method (red line) for the D-F sample. The values with an asterisk (*) are the corresponding ppm error of the peaks in broadband.

4. Space-charge effects in extremely complex samples

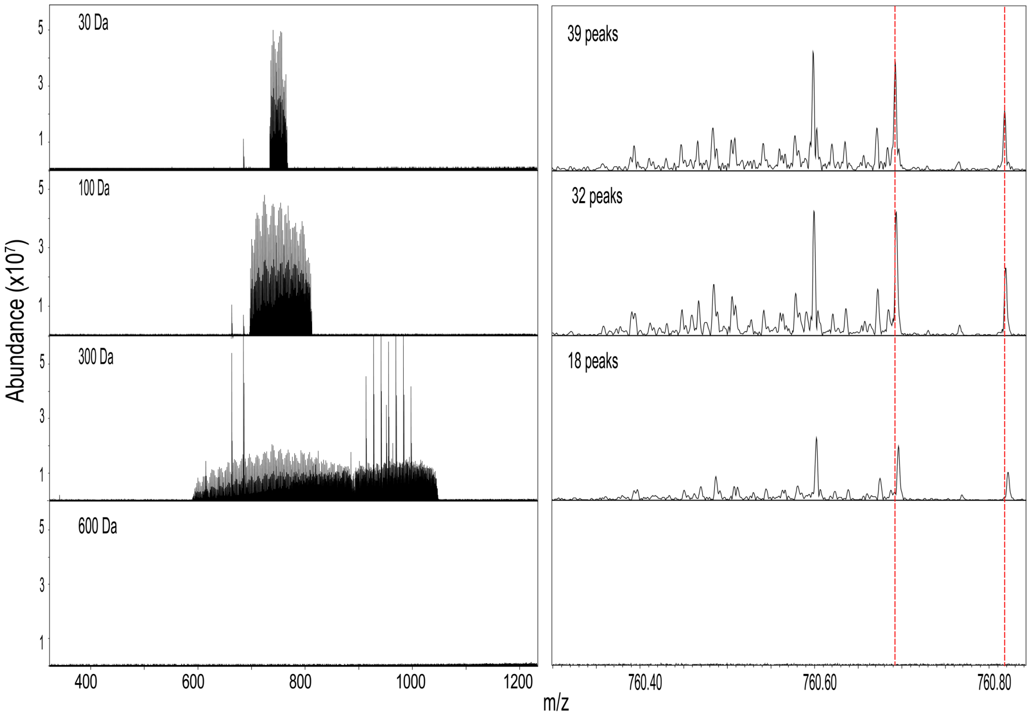

Fig. S14 Mass spectra of the ND-F fraction obtained by positive-ion ESI FT-ICR MS using varying isolation windows sizes. The spectra were obtained with a constant low mass cut-off at *m/z* 200, with an ion accumulation time of 3 seconds. The isolation window center was *m/z* 750 and only the window width was changed (values listed on left hand side of the data sets). Using a window *m/z* width of 600, no signal was observed. The right-hand side shows enlarged *m/z* regions, centered at *m/z* 760. As can be seen, when increasing the window widths, peaks separated by small mass difference tend to coalesce and peaks also shift to higher *m/z*, both as a consequence of multiple space-charge effects due to the increasing number of ions.

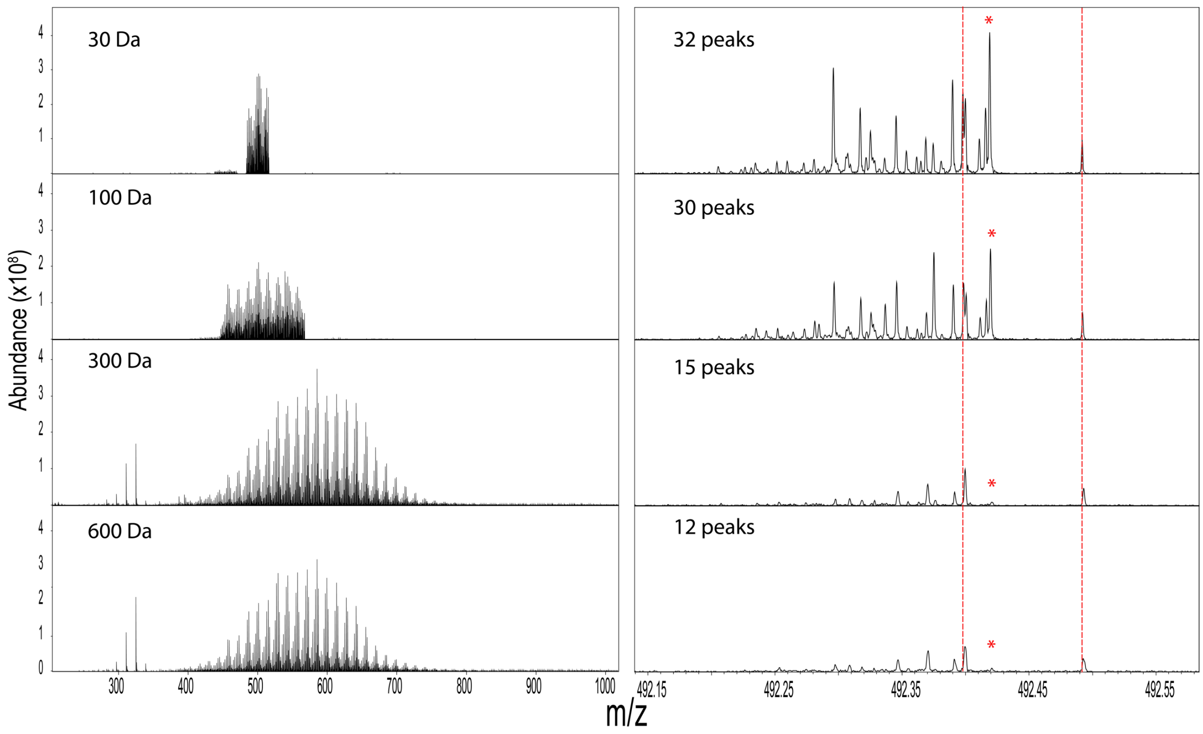

Fig. S15 Mass spectra of the D-F sample obtained by positive-ion ESI FT-ICR MS using varying isolation window sizes. The spectra were obtained with a constant low mass cut-off of *m/z* 200, with an ion accumulation time of 0.8 seconds. The isolation window center was *m/z* 500. In contrast to a very complex sample, such as the ND-F fraction, the windows width not significantly affect the signal intensity. Space-charge effects were observed, however, as a consequence of the increased window widths leading to greater on populations within the instrument.

5. Truly non-distillable sample (ND-F)

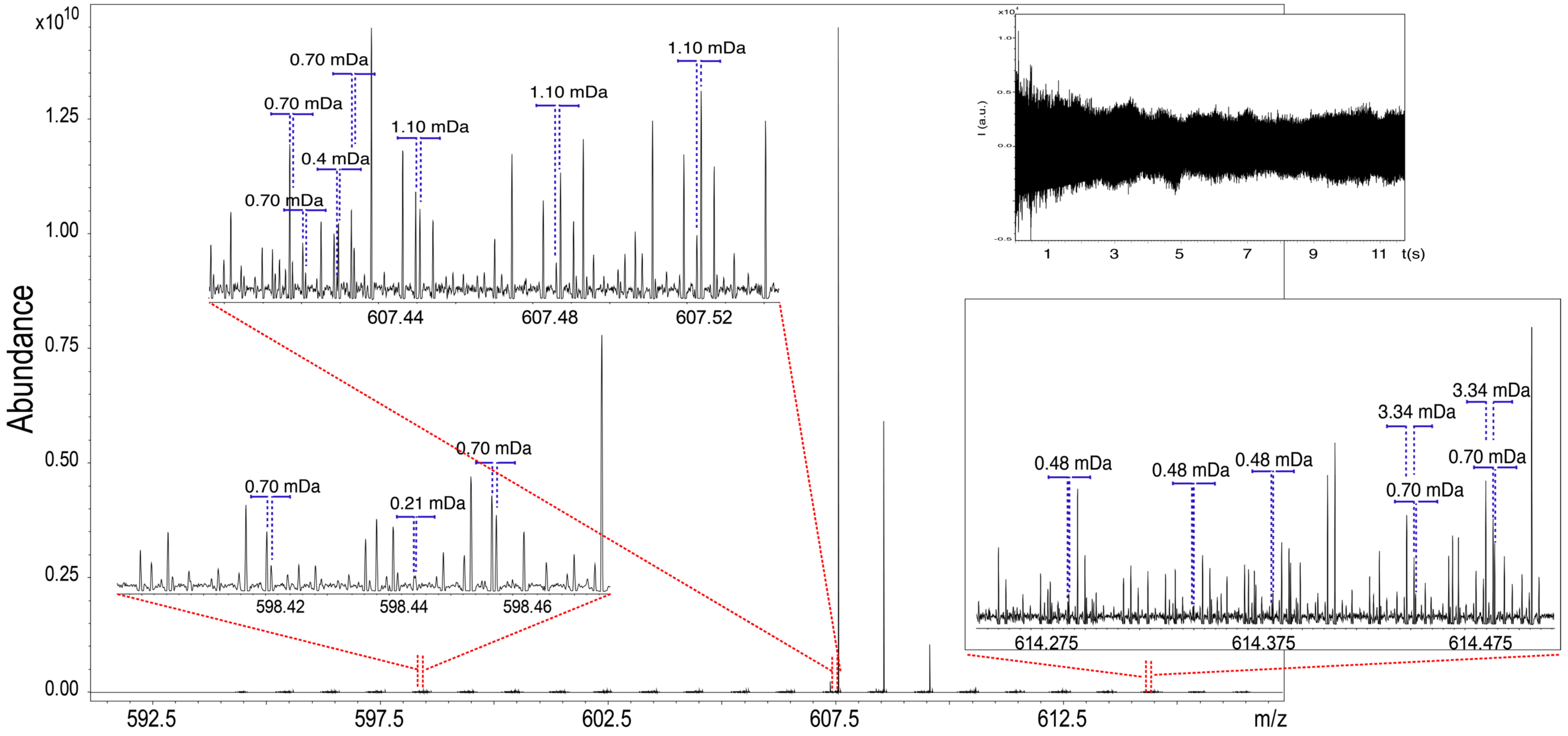

Fig. S16 Mass spectrum acquired using the OCULAR method for the ND-F sample, with a window *m/z* width of 24, centered at *m/z* 605, and with a low mass cut off at *m/z* 515.9. The transient length of approximately 12 s allows a mean resolving power of 3,130,000 FWHM. The enlarged regions show selected mass differences between assigned peaks.

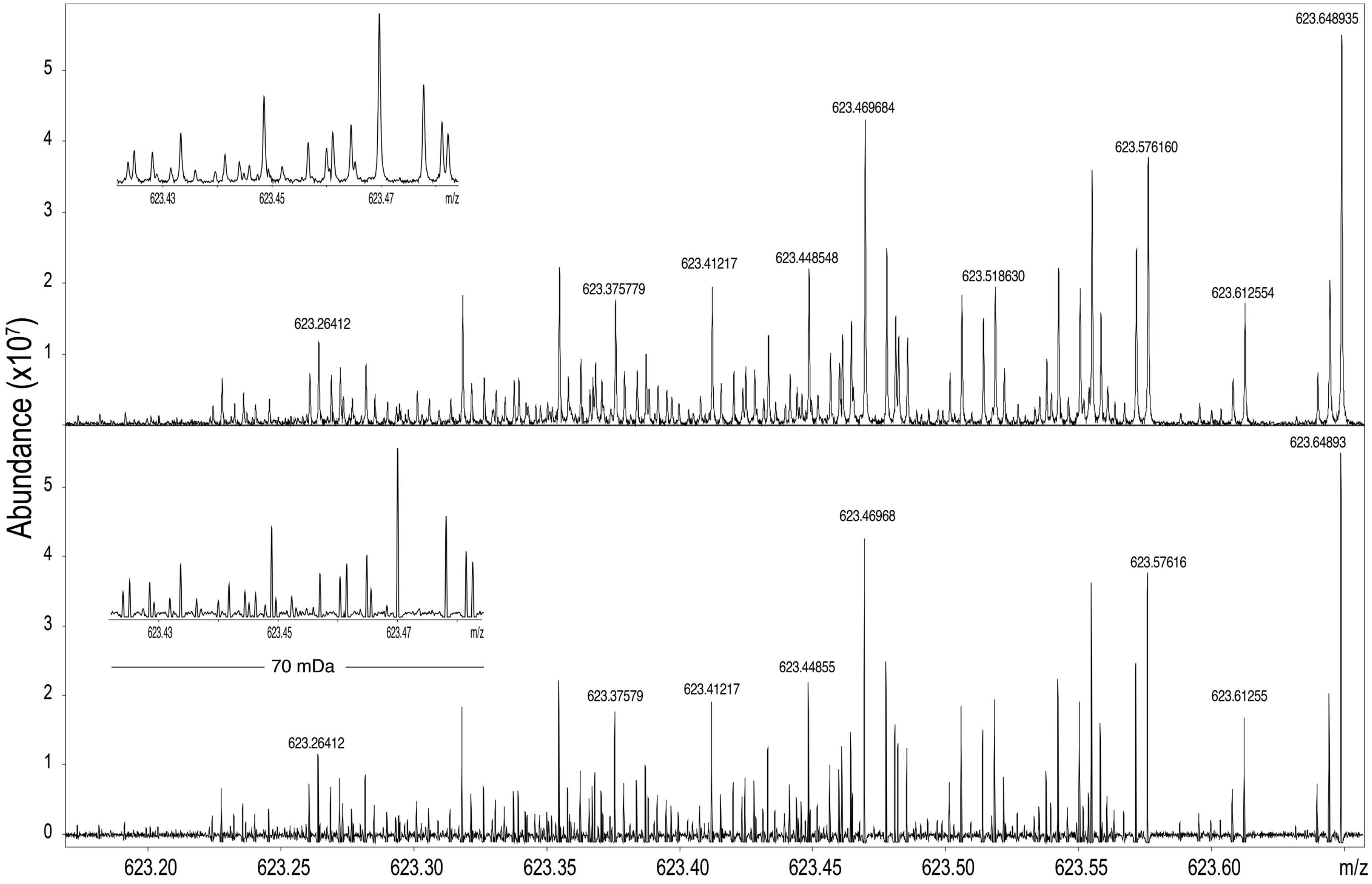

Fig. S17 Comparison between 1 Da regions of segments for *m/z* 623, both in magnitude (above) and absorption mode (bottom), for the ND-F sample. The mean resolving power of 1,800,000 FWHM in magnitude mode was increased 1.7-fold to 3,060,000 FWHM by using absorption mode.

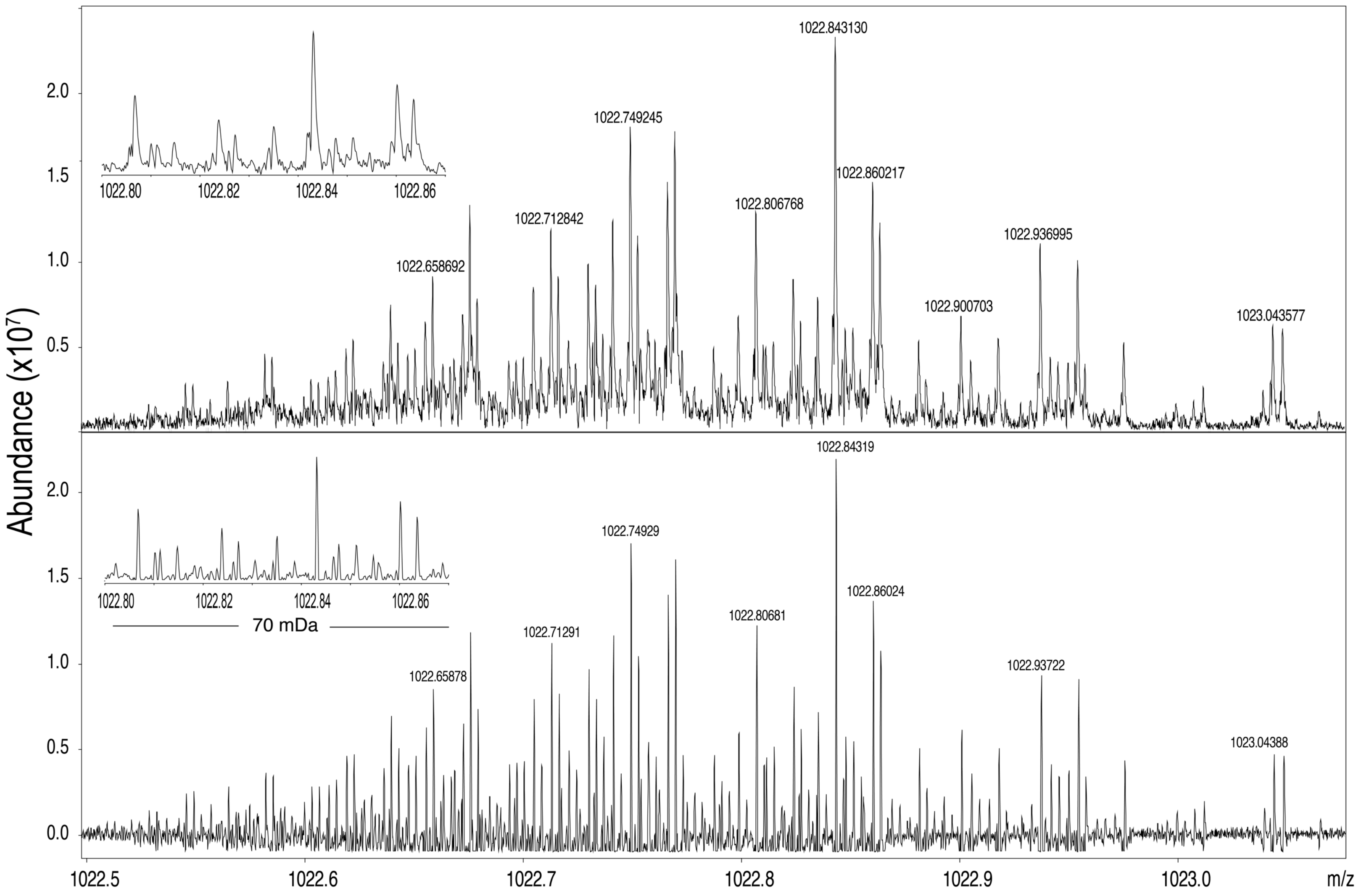

Fig. S18 Comparison between 1 Da regions of segments centred at *m/z* 1022, both in magnitude mode (above) and absorption mode (bottom) for the ND-F sample. The resolution is increased by approximately 1.6x from 1,890,000 FMHW to 3,054,000 FWHM when moving from magnitude mode to absorption mode.

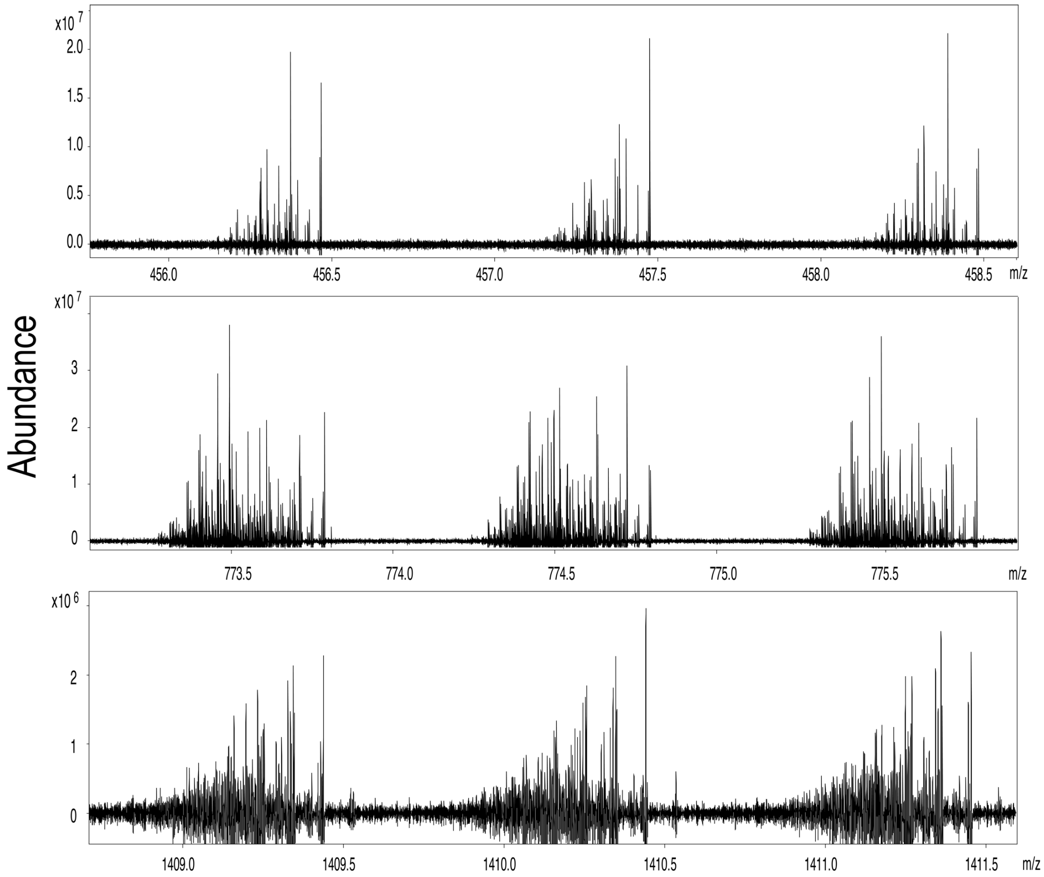

Fig. S19 Comparison of varying mass defect distributions at each nominal mass, spanning different *m/z* ranges, for the ND-F sample. The number of peaks per nominal Da increases with increasing *m/z*.

Table S6

Approximate number of peaks detected with smallest mass separations (below 3.4 mDa and rounded to two decimal places) specified below for the ND-F sample; the differences therefore count each peak once, instead of allowing a peak to have multiple mass separations. The asterisk denotes mass differences reported in previous works.[13]

**Mass difference**		**Number of peaks**
**3.37 mDa***	C_3 vs SH_4	32,903
**1.79 mDa***	$C^{13}C^{14}N$ vs H_3O_3	10,190
**1.10 mDa***	C_4 vs $^{13}CH_3{}^{32}S$	37,651
**0.69 mDa***	C_6N vs H_5O_3S	3,557
**0.56 mDa***	$C^{13}C^{14}N$ vs $H_5{}^{34}S$	1,858
**2.29 mDa**	C_3H_2O vs $^{13}C_2N_2$	4,460
**1.08 mDa**	$^{13}C_2N_2$ vs H_6OS	4,195
**0.96 mDa**	C_4N_2 vs $^{13}C_2H_2O_3$	1,411
**0.83 mDa**	$^{13}C_3$ vs C_2HN	7,245
**0.71 mDa**	C_2H_3S vs $^{13}CNO_2$	10,676
**0.58 mDa**	H_4OS_2 vs $C_3N^{34}S$	281
**0.54 mDa**	$H_8S^{34}S$ vs C_5N	211
**0.45 mDa**	N_2O_2H vs $C_4{}^{13}C$	669
**0.39 mDa**	$^{13}C_2NO_2$ vs C_6	1131

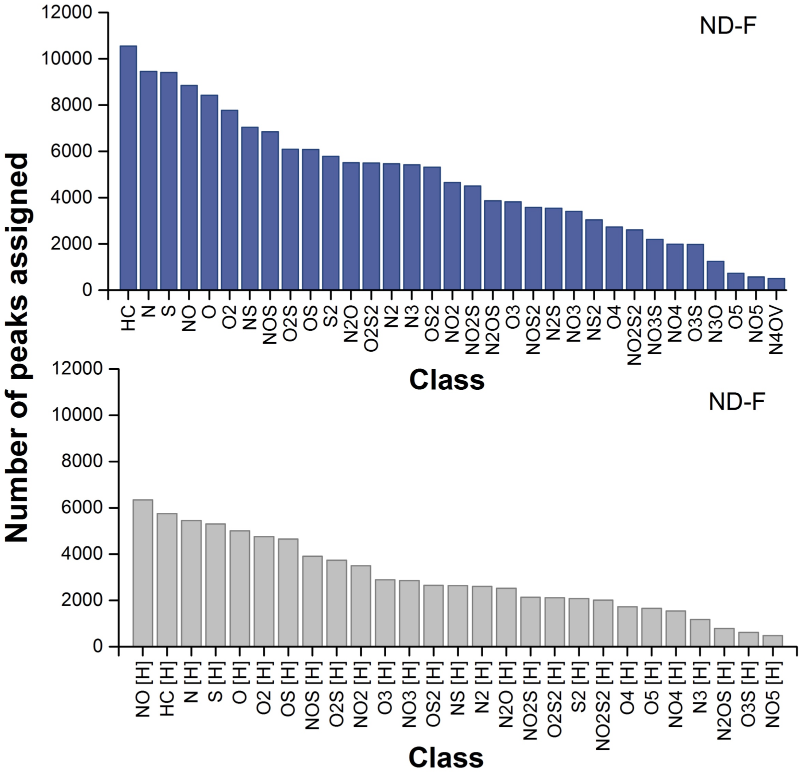

Fig. S20 Number of peaks assigned per class for the OCULAR data set for the ND-F sample. The species with the greatest number of peaks corresponded to less complex heteroatomic compositions. Similarly, to the results obtained for the D-F sample, almost the radical ion species accounted for approximately twice as many peaks as the protonated species.

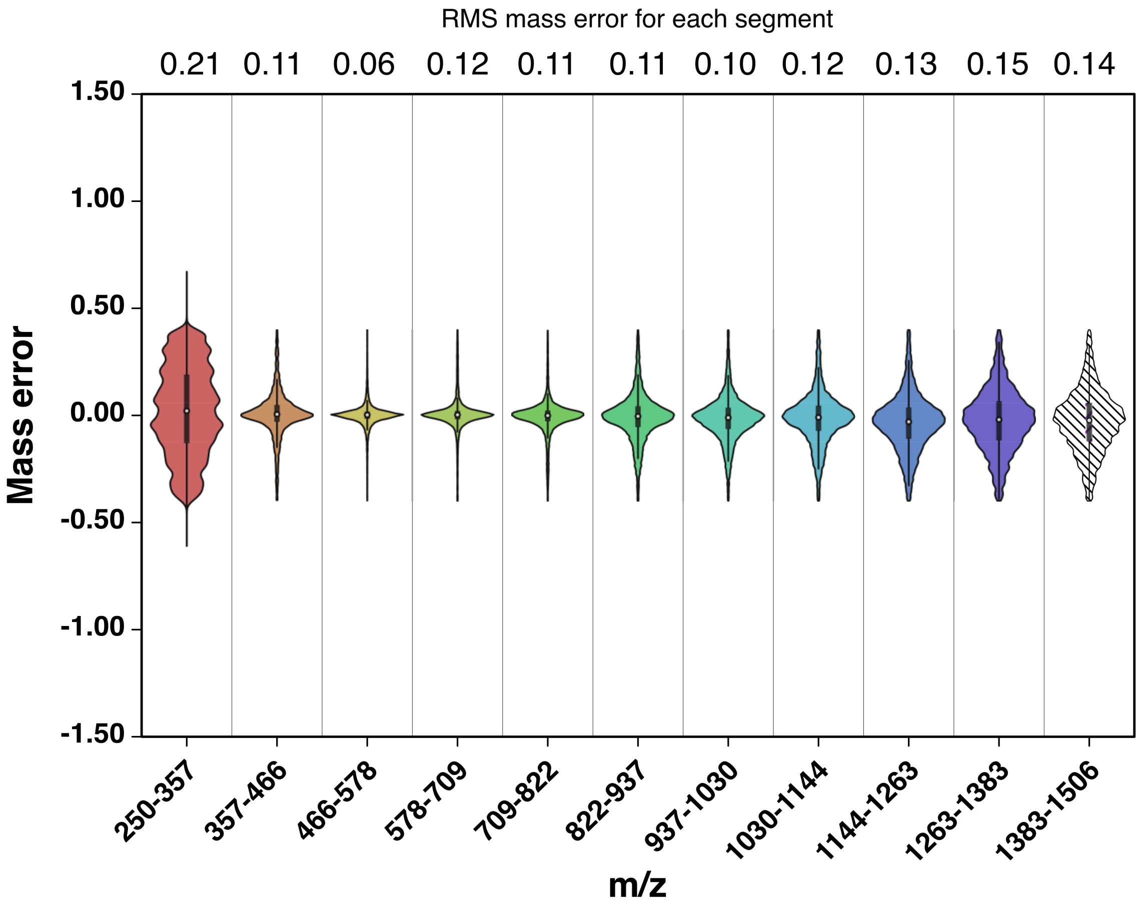

Fig. S21 Violin plots showing the range of mass error (in ppm) of the assignments for the ND-F sample using the OCULAR method. The error is expressed in ppm and the RMS mass errors for each 100 Da segment is listed at the top. The high error found at low *m/z* was due to the combination of the low number of peaks and low signal-to-noise in this region.

References

1 M.-J. Parra, A.-Y. Leon and L. Ja. Hoyos, *CT&F - Ciencia, Tecnol. y Futur.*, 2010, **4**, 83–90.

2 E. R. L. Rocha, M. S. Lopes, M. R. Wolf MacIel, R. MacIel Filho and L. C. Medina, *Ind. Eng. Chem. Res.*, 2013, **52**, 15488–15493.

3 ASTM Standard D4052-16, *Standard Test Method for Density, Relative Density, and API Gravity of Liquids by Digital Density Meter*, ASTM International, West

Conshohocken, PA, 2016.

4 *ASTM D2007 Standard Test Method for Characteristic Groups in Rubber Extender and Processing Oils and Other Petroleum-Derived Oils by the Clay-Gel Absorption Chromatographic Method*, ASTM International, West Conshohocken, PA, 2016.

5 *ASTM D1552 Sulfur in Petroleum Products*, West Conshohocken, PA, ASTM Inter., 2003.

6 S. Golden, T. Barletta and S. White, *Sour & Heavy*, 2012, **1**, 11–15.

7 Y. Qi and P. B. O'Connor, *Mass Spectrom. Rev.*, 2014, **33**, 333–352.

8 E. N. Nikolaev, *Int. J. Mass Spectrom.*, 2015, **377**, 421–431.

9 Y. Cho, A. Ahmed, A. Islam and S. Kim, *Mass Spectrom. Rev.*, 2015, **34**, 248–263.

10 Y. Cho, A. Ahmed and S. Kim, *Anal. Chem.*, 2013, **85**, 9758–9763.

11 L. C. Krajewski, R. P. Rodgers and A. G. Marshall, *Anal. Chem.*, 2017, **89**, 11318–11324.

12 A. Gaspar and W. Schrader, *Rapid Commun. Mass Spectrom.*, 2012, **26**, 1047–1052.

13 D. F. Smith, D. C. Podgorski, R. P. Rodgers, G. T. Blakney and C. L. Hendrickson, *Anal. Chem.*, 2018, **90**, 2041–2047.
